# SENP1 drives glycolysis and cisplatin resistance in gastric cancer via desumoylating ENO1

**DOI:** 10.1186/s13046-025-03543-z

**Published:** 2025-10-08

**Authors:** Yuan Fang, Yunru Gu, Tingting Xu, Peng Wang, Xi Wu, Haoyang Shen, Yangyue Xu, Zixiang Xu, Lei Cao, Xiao Li, Hao Wu, Yongqian Shu, Pei Ma

**Affiliations:** 1 Department of Oncology, The First Affiliated Hospital with Nanjing Medical University, Nanjing, 210029 China; 2 Department of General Surgery, Nanjing Drum Tower Hospital, The Affiliated Hospital of Nanjing University Medical School, Nanjing, 210008 China; 3 Department of Oncology, Wuxi People’s Hospital, Wuxi Medical Center, The Affiliated Wuxi People’s Hospital of Nanjing Medical University, Nanjing Medical University, Wuxi, 214000 China; 4 Department of Oncology, The Affiliated Suqian First People’s Hospital of Nanjing Medical University, Suqian, 223800 Jiangsu China; 5 Department of Pathology, The First Affiliated Hospital of Nanjing Medical University, Nanjing, 210029 China; 6 Jiangsu Key Lab of Cancer Biomarkers, Prevention and Treatment, Collaborative Innovation Center for Cancer Personalized Medicine, Nanjing Medical University, Nanjing, 211166 China; 7 Department of Oncology, Jiangsu Province Hospital Jurong Branch, Zhenjiang, 212400 Jiangsu China

**Keywords:** Gastric cancer, SUMOylation, SENP1, Glycolysis, Cisplatin

## Abstract

**Background:**

Gastric cancer remains a leading cause of cancer-related mortality in world, with advanced-stage patients facing poor prognosis despite emerging therapies. SUMOylation modification is a major post-translation modification, which is essential for cellular behaviors. However, the potential function of SUMOylation in gastric cancer (GC) and the underlying molecular mechanisms remain unclear.

**Methods:**

In our study, a bioinformatics analysis was conducted to screen potential regulators within the SUMO-Specific Peptidase (SENP) family in GC. In vitro functional experiments including CCK8, colony formation, transwell assay, sphere formation, Glycolytic flux, ECAR and OCR and several animal models including GC xenografts, organoids and lung metastasis models were employed to ascertain the role of SENP1 in GC progression and metastasis. Mass spectrometry analysis, coimmunoprecipitation and immunofluorescence staining were performed to elucidate the mechanisms by which SENP1 functions in GC cells.

**Results:**

We identified that SENP1 was upregulated in GC tissues and correlated with a poor prognosis. Multiple functional experiments demonstrated that SENP1 promotes the proliferation, migration, stemness and glycolysis of GC cells. Mechanistically, SENP1 binds to α-enolase (ENO1) and deSUMOylates the SUMO sites (K256, K394) of SUMO2-modified ENO1, enhancing ENO1 stability and drive gastric tumorigenesis. Meanwhile, SENP1 inhibitor Momordin Ιc (Mc) in combination with cisplatin has a synergistic effect on gastric tumor growth in vitro and in vivo.

**Conclusion:**

SENP1 facilitates gastric cancer progression by metabolic reprogramming. Targeting SENP1 with Momordin Ic is a novel therapeutic approach for GC patients.

**Supplementary Information:**

The online version contains supplementary material available at 10.1186/s13046-025-03543-z.

## Background

Gastric cancer (GC) is a highly aggressive malignancy and ranks as the fifth leading cause of cancer-related deaths in China [1, 2]. Despite significant advancements in targeted treatments and immunotherapies for GC, patients continue to face an unfavorable prognosis due to the recurrence and metastasis of the disease following surgery [3–5]. To facilitate the development of effective therapeutic interventions to combat this fatal illness, it is imperative to gain a deeper understanding of the molecular pathways implicated in GC and identify novel molecular targets to enhance the sensibility of cisplatin.

Small ubiquitin-like modifiers (SUMOs) are post-translational modifications (PTMs) involved in various cellular processes. The conjugation of SUMO proteins to substrate proteins, called SUMOylation, is modulated by E1 activating enzyme (SAE1/UBA2), E2 conjugating enzyme (UBC9), E3 ligating enzyme, and sentrin/SUMO-specific proteases (SENP) [6–8]. SENPs play crucial role in cancer progression, including cell cycle progression, the DNA damage response and glycolysis [7].

Several SENP proteins have been recognized as essential regulators in various kinds of cancers. SENP1 is highly expressed in tumors and promotes the proliferation and aggressiveness of breast [9], colon [10], liver [11] and bladder [12] cancers. SENP2 involves in liver [13] and bladder [14] cancer. SENP3 facilitates macrophage polarization in breast cancer [15] and SENP5 is related to differentiation of OSCC [16]. However, the role of SENP in gastric cancer has not been systematically studied.

Known as the Warburg effect, aerobic glycolysis stands as both a hallmark of cancer cells and the foundation for various biological characteristics exhibited by these cells [17]. In numerous types of tumors, the Warburg effect triggers an escalation in total glycolysis, not only under normal oxygen conditions but also in hypoxic conditions [17–19]. Interestingly, SENP1 has been reported to be close to glycolysis in cancer cells [11, 20–22]. HK, PKM2, ENO1, GLUT and et al. are reported to close related to glycolysis [23, 24]. Former studies have revealed that glycolytic enzymes, such as Enolases, play a critical role in the glycolytic processes within cancer cells [25]. ENO1, one of four types of Enolase isozymes, is a glycolytic enzyme that plays essential roles in various pathological activities including cancer development [26, 27]. Furthermore, recent studies have elaborated that the overexpression of ENO1 contributes significantly to the onset and progression of GC. Sun et al. found that ENO1 enhances GC cell proliferation and metastasis through AKT signaling pathway [28]. Qiao et al. demonstrated that suppression of ENO1 increases chemosensitivity of GC cells [29]. There is also increasing evidence that ENO1 increased stemness of GC cells by stimulating glycolysis [30].

In this study, we aimed to reveal the biological and clinical significance of SENP1 in GC. Molecular biology studies uncovered that SENP1 interacted with ENO1 and SENP1 mediated the deSUMOylation of ENO1, which inhibited ENO1 proteasome-mediated degradation. Furthermore, the results of pharmacological inhibition of SENP1 suggested a potential therapeutic strategy to overcome chemoresistance. In summary, our findings demonstrated the critical role of the SENP1/ENO1 axis in GC progression. Inhibiting this signaling axis enhanced the anti-tumor effects of cisplatin, as evidenced by preclinical models of GC therapeutic action.

## Materials and methods

### Specimens

We collected the GC tissues and adjacent normal gastric tissues from patients with GC. All the patients with GC needed surgical treatment and hospitalized in Affiliated People’s Hospital of Jiangsu University. The study received ethical approval from Nanjing Medical University (2018-SRFA-074) and Jiangsu University Affiliated People’s Hospital (K20180016Y), which was implemented in accordance with the Helsinki Declaration of Principles. All gastric tissues and paired adjacent tissues were surgically removed and used for RNA and protein extraction and immunohistochemical (IHC) analysis.

### Bioinformatic analysis

The RNA sequencing data and curated survival data of STAD was downloaded from the database of GSE27342 (*n* = 80) [31], and GSE56807 (*n* = 5) [32]. Differential analysis identified genes with a fold-change over 1.5. We used the survminer R package to determine the optimal cutoff value. Two bioinformatics websites, UCSC genome browser and GSE162420 [33], was used to analyze the promoter region of SENP1. Differential analysis was performed using the Diffbind package. The gene set GSE183904 [34] was selected to analyze the relationship between SENP1 and glycolysis in GC, which included a total of 31 primary GC samples and 9 normal tissue samples, for the annotation of single-cell RNA sequencing results.

### Cell lines and cell culture

The human GC cell lines (include MKN-45, HGC-27, AGS) and normal human gastric epithelial cell line GES-1 were purchased from Shanghai Cell Bank Library. All cell lines were tested negative for mycoplasma contamination and were authenticated by STR profiling and flow cytometry. RPMI 1640 (Gibco, Carlsbad, CA, USA) supplemented with 10% FBS (BI, Israel) and 1% penicillin-streptomycin (Invitrogen, USA) was used to culture all cells. All the cells were culture at 37◦C under 5% CO_2_ conditions.

### Antibodies and reagents

Details of the antibodies used for IHC are shown in Table S3. The Protein A/G PLUS-Agarose (sc-2003) was purchased from Santa Cruz Biotechnology (USA). The anti-Ki67 (GB121141-100) antibody used to IHC staining and Fluorescein (FITC) Tunel Cell Apoptosis Detection Kit used to tissue immunofluorescence assay were purchased from servicebio (China). The MG132 (HY-13259) and Cycloheximide (CHX, HY-12320) used to Ubiquitination assay were purchased from MCE corporation. The cisplatin (S1166) was obtained from Selleck Chemicals.

### Lentivirus and plasmid

Lentivirus overexpressing SENP1, lentivirus overexpressing wild type ENO1, lentivirus overexpressing two-site mutant ENO1 (K394R, K256R) and lentivirus containing short hairpin RNAs (shRNAs) which targeting SENP1, were made by Genechem corporation (China). The sequence of shRNA used to knockdown SENP1 is listed in Table S2. The pc-DNA3.1-Flag-SENP3, HA-ENO1, HIS-SUMO1, HIS-SUMO2, HIS-SUMO3, RH-SUMO2, HIS-UB, HA-ENO1 (K394R), HA-ENO1 (K256R), HA-ENO1-DKR (K394R, K256R) plasmids were synthesized by Genechem corporation. The Lipofectamine 3000 (Invitrogen, USA) was used to transfect plasmids into HEK293T cells or GC cells.

### Growth proliferation and transwell assay

To detect the proliferation ability of GC cells, we seeded 1000 cells in 96-well plates firstly, the Cell Counting Kit-8 solution was added into culture medium and incubated for 2 h, and OD450 was detected by Multiskan GO (Thermo Fisher).

To exam the invasion ability of GC cells, we added 200ul FBS-free medium containing 10,000 GC cells into the upper region of transwell chamber, then 600ul culture medium with 10% FBS was added into the lower level of transwell chamber. After 24–48 h, the number of cells on the bottom floor of the chamber was detect by crystal violet staining.

### PER, OCR assays

For PER analysis, we seeded cells on poly-lysine coated XFe96 plates firstly, then the samples were subjected to Seahorse XF96 Extracellular Flux Analyzer (Seahorse Bioscience, USA). And baseline was detected firstly, then rotenone and antimycin A (Rot/AA) (0.5µmol/L) and 2-DG (50nmol/L) were added into plates in order at indicated points, all the glycolytic proton efflux rate were detected at different time. For OCR analysis, the cells were seeded as above described. Basal respiration was detected firstly, then Oligomycin (1 µM), FCCP (2 µM) and Rot/AA (1 µM) was added in order according to manufacturer’s instruction. The Oxygen Consumption Rate was detected at different time. The seahorse XF Glycolysis Stress Test Report Generator and Seahorse XF Cell Mito Stress Test Report Generator were used to analyze all the data.

### Pharmaceuticals

The p300 inhibitor C646 was purchased from MCE (Medchem Express, USA). An appropriate number of cells was seeded in 6-well plates before media containing 20 µM of C646 was added to the culture. After 24 h, the cells were collected, and the total mRNA and protein was isolated. For deacetylase inhibitor treatment, SAHA, ACY-1215, TMP195 and MGCD0103 (MedChemExpress, USA) were added to the culture medium of HGC-27 and MKN-45 cells for 48 h at the indicated concentrations.

### Chromatin immunoprecipitation assay (ChIP)

To separate Chromatin binds DNA, the Pierce™ Magnetic ChIP Kit (26157) was purchased from Thermo Fisher (USA). First, the GC cells cultured in plates were fixed with 1% formaldehyde for 10 min at 37℃. Then, glycine solution was used to terminate the Process of fixation. The lysis buffer containing protease inhibitor was used to lyse cells for 10 min. And the cell lysates were subject to ultrasonic processing for acquiring DNA that lengths between 100 and 1000 bp. Next, the cell lysates were incubated with anti-p300 (CST, #54062) or anti- H3K27ac (CST, #8173) on a vertical shaker overnight at 4 °C. The next day, the cell lysates were incubated with protein A/G magnetic beads for 1 h. After washing process, elution buffer was used to extract DNA that binds to histone according to the instruction. The qRT-PCR and DNA agarose gel electrophoresis were conducted to analyze the target DNA. The primers used in this experiment were listed in the Table S1.

### Sphere formation assays

To detect the stemness of GC cells, the MKN-45 and HGC-27 (RRID: CVCL_1279) cells were seeded into 6-well ultralow attachment plates (Corning, USA) and cultured with 1640 medium (includes EGF (20 ng/ml), bFGF (10 ng/ml) and B27 (2%)) without FBS. The status of spheroid formation was observed daily and photographed under a microscope to record.

### Immunoblotting and immunoprecipitation

The protein was extracted from tissues and cells by using RIPA Lysis Buffer (Beyotime, shanghai) with PMSF (1mM, Beyotime, shanghai). The lysis was added with SDS-PAGE Sample Loading Buffer and boiled for 5 min. Next, the protein samples were subjected to SDS-PAGE electrophoresis and Transmembrane. Then, the membrane was incubated with primary antibodies and second antibodies. Last, the chemiluminescence imaging system was used to detect signal in membrane.

For immunoprecipitation assay, the protein was obtained as above described. The antibodies were added into lysis and incubated overnight. Next, Protein A/G PLUS-Agarose (Santa cruz, USA) was added into lysis to bind antibody. Then we obtained agarose contained antibody and special protein by centrifugation. After adding loading buffer and boiling, the sample were subjected to immunoblotting.

### Mass spectrometry

Co-immunoprecipitation assays were performed first. Then the proteins were subjected to SDS-PAGE. Next, the gels that containing protein were subjected to silver staining to confirm the existence of proteins. Last, the Applied protein technology corporation China, Shanghai) performed the analysis of Mass spectrometry in gels that we send.

### RT-qPCR analysis

The Trizol (Invitrogen, USA) was used to extract RNA from tissues or cancer cells as previous described [35]. Reverse Transcriptase purchased from Vazyme (Nanjing) was used to transfer RNA to cDNA. The ChamQ SYBR qPCR Master Mix (Vazyme, Nanjing) was used to detect the relative mRNA expression according to according to manufacturer instructions. All the primer pairs were synthesized by genscript corporation (Nanjing). The sequences of primer pairs were shown in Table S1.

### Immunohistochemistry (IHC) analysis and Multiplex immunohistochemical (mIHC)

For mIHC, 3%H_2_O_2_ was used to restrain endoperoxidase activity firstly. Then, the tissue slide was incubated with primary antibody for 60 min at air wet tank, after washing with TBST, the second antibody (#8114 or HRP mouse, #8125) was used to incubate with tissue slide for 30 min. Next, the tissue slide was incubated with Fluorophore-conjugated TSA^®^ Plus Amplification Reagent according to the manufacturer instructions. Use a microwave oven and boil the slide in a pH 6.0 10 mM sodium citrate buffer. Keep at sub boiling temperature for 10 min. Place the slide on the table and cool to room temperature for 30 min. Repeat the staining process for each primary antibody. Lastly, the DAPI working solution was used to incubate with tissue slide. The Aperio VERSA was used to detect all the signal.

### Animal experiments

For GC cells xenograft mouse model, the HGC-27, MKN-45 and AGS cells (5 × 10^6^) were injected subcutaneously into 5-week-old BALB/c nude male mice. The tumor volume (V = 0.5×length×width^2^) was monitored every 3 days. Six weeks after the model was established, all the mice were sacrificed.

For lung metastasis model, the GC cells were infected with lentivirus expressing luciferase, then 10^6^ tumor cells were injected to 5-week-old BALB/c nude male mice through tail veil.

For drug therapy, the cisplatin or Momordin Ic was administered to mice by intraperitoneal injection.

All the mice were purchased from Gempharmatech (China, Nanjing). And the mice were housed in a specific-pathogen-free (SPF) facility and provided plenty of water and food. Animals were assigned to experimental groups using simple randomization. All the animal experiment protocol were approved Nanjing Medical University’s Committee on Ethics for Animal Experiments (IACUC-1706007).

### Ubiquitination assay

The different plasmids were transfected into HEK293T cells firstly. Then, the protease inhibitor MG132 (10µM) was used to treat cells for 6 h before co- immunoprecipitation. As previous described, we extracted the protein by using lysis buffer with PMSF and protease inhibitor. The anti-HA antibody was used to pull down HA-tagged ENO1 in lysates. And western blot analysis was used to detect ubiquitylation.

### Glucose consumption and lactate production assay

To detect the glucose consumption of GC cells, the cells were cultured in six-well plates for 24 h firstly. Then, the supernatant was collected, and we measured the level of glucose in the medium by using a Glucose Assay Kit (S0201M).

To examine the level of Lactate production, L-Lactate Assay Kit (ab65330) was purchased from Abcam corporation (USA). The Lactate level in the cell culture supernatant was detected according to the manufacturer’s Instructions.

### Measurement of ROS

The cells were washed with PBS three times firstly, then the cells were treated with serum free medium supplemented with DCFH-DA (1:1000, Beyotime, shanghai) for 20 min at 37 °C. Next, the serum free medium was used to wash Residual DCFH-DA. All the cells were harvested and subject to flow cytometry analysis.

### ENO1 activity assay

To detect the activity of ENO1 in GC cells, a ENO1 Assay kit was purchased from Abcam (ab117994, USA). Briefly, the cells were harvested and solubilized by Extraction Buffer for 20 min on ice. After centrifugation, the supernatants were collected for further analysis. The diluted test samples were added into the well of plate in the kit and incubated for 2 h. After washing, each well was added into 200 µL 1X Activity Solution. When 0.15 mmol/L NADH was introduced to initiate the reaction, the absorbance at 340 nm (OD340) decreased, which means the reduction of NADH, the OD340 was recorded by a microplate reader every minute until the end of the one-hour reaction.

### Metabolic flux analysis

The cells were cultured with the specifical medium which containing [U-^13^C] glucose (Cambridge Isotope Laboratories, CLM-1396-1) in six-well plate for 24 h. Then, unlabeled (M + 0) and labeled (M + n) forms of metabolites were detected simultaneously by a targeted metabolomics approach in Metabo-Profile (Shanghai, China) analysis. The mass distribution vector (MDV) was used to describe the proportion of each isotope distribution of the metabolites.

### Chemosensitivity assay

The cells were cultured in 96-well plates for 24 h. Then the cells were rechanged with fresh medium containing different concentrations of cisplatin. The cell viability every day was detected by using CCK8 assay.

### Statistical analysis

The GraphPad Prism 8.0 was used to perform all the statistical tests, and *p* value < 0.05 was considered statistical significance (*p < 0.05; **p < 0.01; ***p < 0.001, ****p < 0.0001). The unpaired two-tailed Student’s t-test was used to analyze differences between two groups. One-way or two-way analysis of variance (ANOVA) was used to analyze differences between multiple groups, and the log-rank test was used to analyze differences in survival curves. The data were presented as mean ± SD, and n values are expressed in the figure legends.

## Results

### SENP1 is highly expressed in clinical gastric cancer tissues and correlates with poor prognosis

**Fig. 1:**
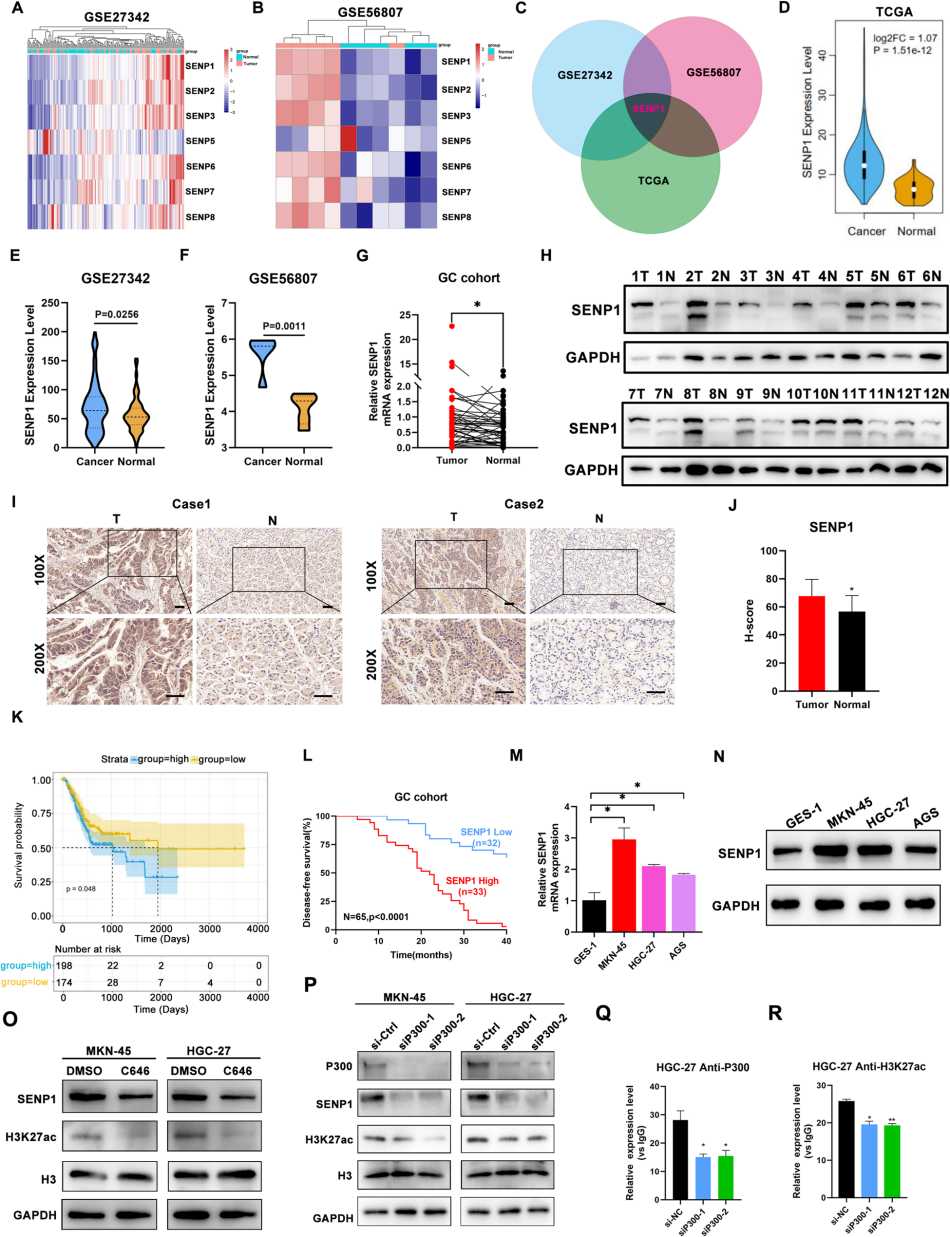
SENP1 is upregulated in gastric cancer tissues and correlates with poor prognosis. **A-B** Heatmaps of SENP1 levels in STAD datasets GSE27342 and GSE56807. **C** Venn diagram showing the most highly expressed genes in the SENP family according to TCGA, GSE27342 and GSE56807. **D** TCGA (The Cancer Genome Atlas) GC database demonstrated that SENP1 was upregulated in GC tumor samples compared with normal samples. **E-F** Box plot analysis of SENP1 mRNA levels in gastric cancer (GC) tissues and normal tissues from the GSE27342 (**E**) and GSE56807 (**F**) datasets. **G** The levels of SENP1 expression in GC and matched adjacent gastric tissues were detected by quantitative real-time polymerase chain reaction (qRT-PCR) (*n* = 65). The PCR results were normalized to the expression of GAPDH. **H** SENP1 protein levels were detected in GC tissues (T) and matched adjacent gastric tissues (N) by western blotting (*n* = 12). Relative protein levels were normalized to GAPDH. **I-J** Representative immunohistochemistry (IHC) staining showed SENP1-positive stained cells in GC tissues and matched adjacent gastric tissues Scale bars,50 μm. **K** Data from the TCGA study showed that progression free interval (PFI) of patients with SENP1 high expression were shorter. **L** Kaplan–Meier analysis revealed disease free survival (DFS) in GC patients based on the relative SENP1 expression (SENP1-High, *n* = 33; SENP1-Low, *n* = 32). **M-N** SENP1 mRNA and protein expression levels in GES-1, MKN-45, HGC-27 and AGS shown by qRT-PCR and WB. **O** The SENP1 and H3K27ac protein levels in MKN-45 and HGC-27 cells were measured by western blotting after C646 treatment (20 µM) for 24 h. **P** The SENP1 and H3K27ac protein levels after p300 knockdown in MKN-45 and HGC-27 cells were determined by western blotting. **Q-R** ChIP assays were used to determine the level of p300 binding (**Q**) and the enrichment of H3K27ac (**R**) at the promoter of SENP1 in p300 deficiency or control HGC-27 cells. *p < 0.05; **p < 0.01; ***p < 0.001

Using data from The Cancer Genome Atlas (TCGA) and Gene Expression Omnibus (GEO), differential expression of SENP genes was identified between normal gastric tissue and GC tissues. Among all SENP family members, we found that only SENP1 expression was upregulated in all three different datasets (Fig. 1A-F). In lines with these transcriptomic results, we observed that SENP1 RNA and protein levels were increased in our GC cohort (Fig. 1G-H). We also detected the SENP1 expression via Immunohistochemistry (IHC) staining. As shown in Fig. 1I-J, SENP1 expression was

higher in GC tissues than in normal tissues. Besides, Kaplan–Meier analysis revealed that SENP1 was negatively associated with the progression free interval (PFI) in GC patients in TCGA database (Fig. 1K). Patients with lower IHC scores showed a favorable prognosis in comparison with their counterparts in our GC cohort (Fig. 1L). In addition, compared to normal gastric epithelial cell line GES1, upregulated SENP1 protein and mRNA expression was observed in three human GC lines (including MKN-45, HGC-27, AGS) (Fig. 1M and N). These data support an important role for SENP1 in GC development.

### p300-mediated H3K27ac activates SENP1 transcription in GC

Recent studies have shown that the potential modification of H3K27ac in the promoter of oncogenes in GC could upregulate their level [36, 37]. We used two bioinformatics websites, UCSC genome browser and GSE162420 [33], to analyse the promoter region of SENP1. Notably, a significant abundance of H3K27ac was observed at the promoter region of SENP1 in GC cells. (Supplementary Fig. 1A-B). H3K27ac is reported to be catalyzed by the p300/CBP complex and HDACs [37, 38]. We used C646 (a histone acetyltransferase inhibitor targeting p300) and other HDACs inhibitor according to our previous research [39]. we observed that the mRNA and protein levels of SENP1 were only decreased by using C646 in GC cells (Supplementary Fig. 1C-D). Furthermore, using C646 in GC cells generated to the reduced level of SENP1 and H3K27ac. (Fig. 1O). We used siRNAs to target HDACs and similarly found that when HDACs were reduced, the expression of SENP1 in GC remained unchanged (Supplementary Fig. 1E-F). However, SENP1 was downregulated when p300 was reduced (Supplementary Fig. 1G).

We also used siRNA to knockdown p300 to explore the effect of H3K27ac. Decreased expression of H3K27ac was observed after p300 knockdown (Fig. 1P). Moreover, we performed the chromatin H3K27ac immunoprecipitation (ChIP) assay by using p300 antibody and H3K27ac antibody. The results of ChIP assay indicated that p300 and H3K27ac could bind to the promoter region of SENP1, the enrichment of SENP1 promoter in H3K27ac was modulated by p300 (Fig. 1Q-R, Supplementary Fig. 1H-I). Together, these data showed that p300-mediated H3K27ac activates SENP1 transcription in GC, which ultimately contributes to high SENP1 expression in GC progression.

### SENP1 exerts oncogenic effects in gastric cancer

**Fig. 2:**
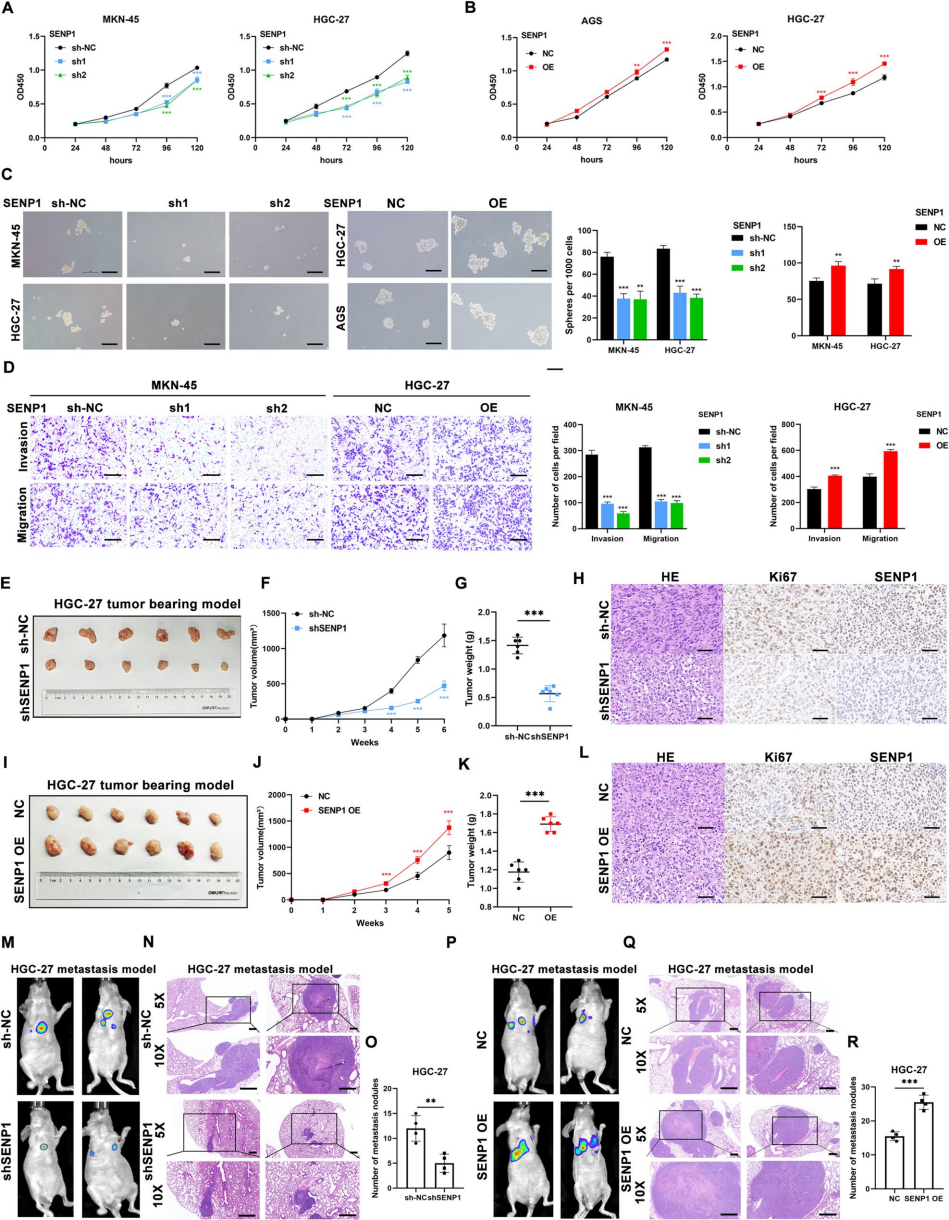
SENP1 promotes gastric cancer cell growth and migration. **A-B** CCK-8 assay of MKN-45, HGC-27 (**A**) and AGS, HGC-27 (**B**) cells transfected as indicated. **C** Representative images of formatted spheres among indicated cells. Scale bar, 100 μm. **D** Cell invasion and migration assays of GC cells transfected as indicated. Scale bar, 500 μm. **E** The images of dissected tumors from HGC-27 cells stably transfected with sh-NC and sh-SENP1. **F-G** Tumor weights and sizes are represented as means of tumor weights (**G**)/sizes (**F**) ± standard deviation (SD). **H** Tumor tissue samples were immunostained for haematoxylin and eosin (H&E), Ki‐67 and SENP1. Scale bar, 50 μm. **I** The images of dissected tumors from HGC-27 cells stably transfected with NC and SENP1 OE. **J-K** Tumor weights and sizes are represented as means of tumor weights (**K**)/sizes (**J**) ± standard deviation (SD). **L** Tumor tissue samples were immunostained for haematoxylin and eosin (H&E), Ki‐67 and SENP1. Scale bar, 50 μm.**M-O** Representative images of bioluminescent images of mice by IVIS Imaging system and HE staining of lung tissues from HGC-27 cells stably transfected with sh‐NC and sh-SENP1. Knockdown of SENP1 suppressed GC lung metastasis in nude mice. Scale bar, 500 μm. **P-R** Representative images of bioluminescent images of mice by IVIS Imaging system and HE staining of lung tissues from HGC-27 cells stably transfected with NC and SENP1 OE. Overexpression of SENP1 upregulated GC lung metastasis in nude mice. Scale bar, 500 μm. *p < 0.05; **p < 0.01; ***p < 0.001

To evaluate the biological function of SENP1 in GC, we performed series of gain- and loss-of-function studies. SENP1 knockdown HGC27 and MKN-45 cells and SENP1 overexpression HGC-27 and AGS cells were established (Supplementary Fig. 2A, 2B). Silencing SENP1 resulted in reduced proliferation of HGC27 and MKN-45 cells, while overexpressed SENP1 promoted the GC cells proliferation (Fig. 2A-B). Given that SENP1 has been identified as a positive regulator of hepatocellular carcinoma (HCC) stemness [11], we assessed the stemness feature of GC cells after SENP1 knockdown/overexpression by using sphere formation assay. As shown in Fig. 2C, SENP1 knockdown resulted in formative spheres much smaller and fewer compared to normal control, which lead to the opposite results while SENP1 overexpression. Consistently, the protein levels of cancer stem cell markers CD44, Nanog, Oct4, and SOX2 were upregulated by SENP1 (Supplementary Fig. 2C-D). Besides, we found that SENP1 promoted the invasion and migration of GC cells, as indicated by Transwell assays (Fig. 2D, Supplementary Fig. 3A, 3B). Furthermore, the impact of SENP1 on the in vivo tumor growth of GC xenografts was investigated. SENP1 knockdown significantly suppressed the tumor growth (Fig. 2E-G, Supplementary Fig. 3C-E). Furthermore, the immunohistochemistry of Ki67 demonstrated that SENP1 inhibited GC cell proliferation in vivo (Fig. 2H, Supplementary Fig. 3F). However, SENP1 overexpression promoted the tumor growth in xenograft mouse model (Fig. 2I-K, Supplementary Fig. 3G, 3I, 3J). Also, more Ki67 positive staining cells in SENP1 overexpression group was observed compared with that in control group (Fig. 2L, Supplementary Fig. 3H). To investigate the effect of SENP1 in metastasis of GC cells in vivo, we used lung metastasis model by tail vein injection of GC cells. The results showed that SENP1 knockdown decreased the incidence of lung metastasis (Fig. 2M-O). Whereas SENP1 overexpression strengthened the invasion and migration of HGC-27 cells in vivo (Fig. 2P-R).

Previous studies have shown that SENP1 is closely associated with glycolysis, which is one of the most important malignant phenotypes of tumors [20, 21, 40, 41]. To validate the correlations of SENP1 with glycolysis, the gene set GSE183904 [34] was selected, including a total of 31 cases of primary GC and 9 normal tissues, for single-cell sequencing result annotation. In gastric cancer, high expression of SENP1 was positively correlated with glycolysis activation (Supplementary Fig. 4A-B). We next designed a series of experiments to validate the relationship of SENP1 and glycolysis in GC. SENP1 downregulation reduced glucose consumption and lactate acid production and overexpressed SENP1 increased glucose consumption and lactate acid production (Supplementary Fig. 4C-F). Reactive oxygen species (ROS) are viewed as an inevitable byproduct of mitochondrial oxidative phosphorylation. Glycolysis of tumor cells reduces ROS to protect the normal function of mitochondria [20]. Therefore, we used a flow cytometer assay to quantify intracellular ROS levels. Compared with their vector controls, SENP1 knockdown GC cells exhibited an increased ROS level (Supplementary Fig. 4G). Similarly, alterations of PER/OCR indicated that SENP1 knockdown impaired glycolytic reserves and promoted spare respiratory capacity (Supplementary Fig. 4H-K). SENP1 overexpression promoted glycolytic reserves and impaired spare respiratory capacity (Supplementary Fig. 4L-O). To examine the direct effect of SENP1 on glycolysis, we performed a metabolic flux analysis in GC using [U-^13^C] glucose as a substrate. Metabolic tracking revealed a significantly increased abundance of glycolysis metabolite M3 PEP, M3 pyruvate and M3 lactate in the SENP1-overexpressing group compared with the NC group, but not TCA-circulating metabolites such as M2 citrate, M2 glutamine, M2 succinate, M2 fumarate, and M2 malate (Supplementary Fig. 4P-Q). Taken together, these data indicated that SENP1 plays a pivotal role in the proliferation, metastasis, maintenance of the stem cell-like characteristics and glycolysis of GC cells.

### SENP1 directly interacts with ENO1

**Fig. 3:**
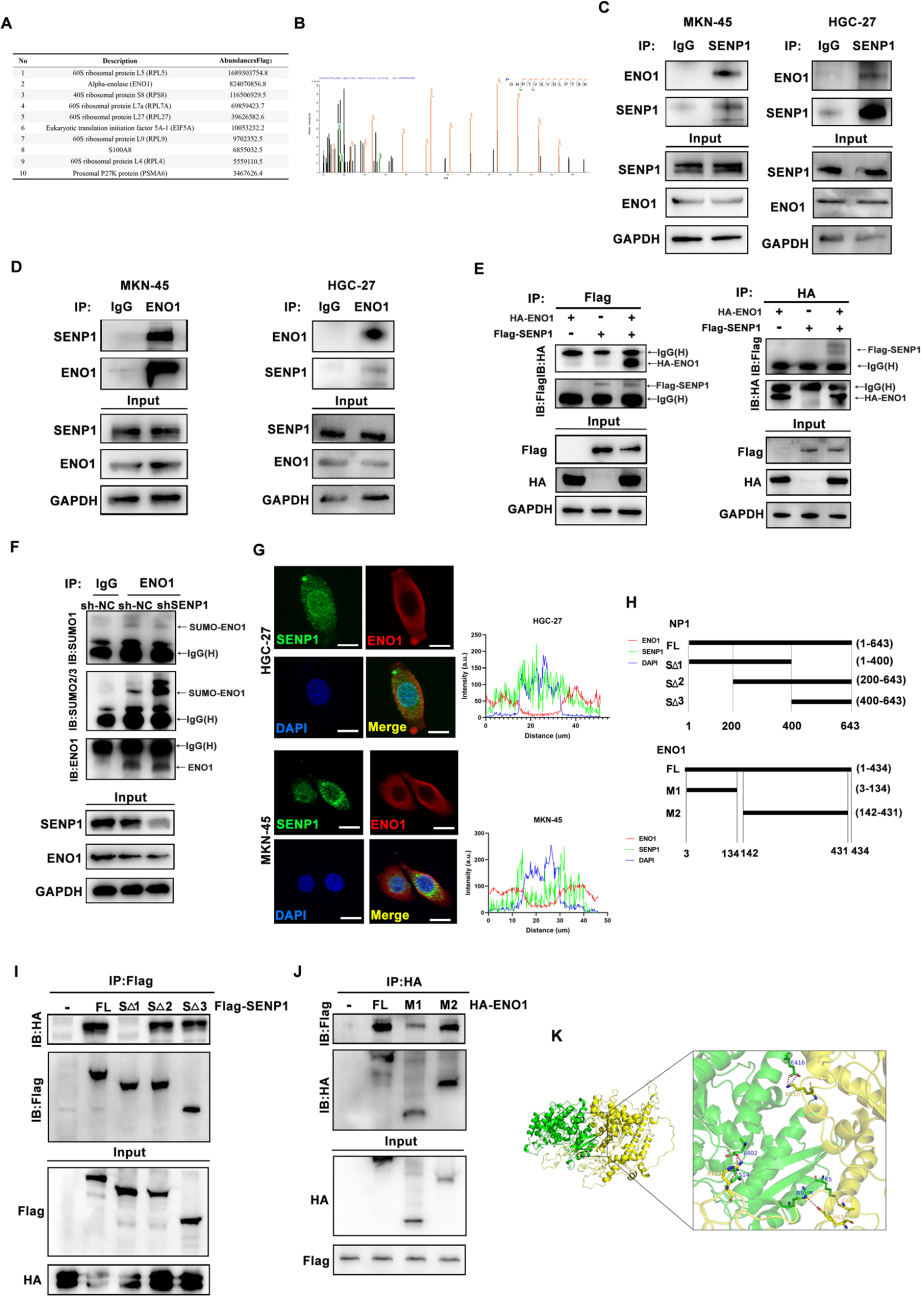
SENP1 directly interacts with ENO1. **A** List of the top 10 proteins with the highest abundance value by mass spectrometry. **B** The peak of ENO1 identified via MS analysis. **C-D** Co-IP validation of the interaction between SENP1 and ENO1 in MKN-45 and HGC-27. **E** Co-IP validation of the interaction between SENP1-Flag and ENO1-HA. **F** Co-IP showing that SUMO2/3, but not SUMO1, was reduced after SENP1 downregulation. **G** Immunofluorescence images showing the distribution of SENP1 (green) and ENO1 (red) in MKN-45 and HGC-27 cells. Scale bar, 10 μm. **H** Schematic structures of SENP1 and ENO1, together with their truncated mutants. **I** Co-IP showing that the S1 of SENP1 is essential for the interaction with ENO1. **J** Co-IP shows that the C terminal of ENO1 is indispensable for the interaction with SENP1. **K** Molecular-docking assays between SENP1 and ENO1. *p < 0.05; **p < 0.01; ***p < 0.001

To elucidate how SENP1 promotes GC progression by affecting glycolysis, we used immunoprecipitation (IP) and mass spectrometry to identify SENP1-interacting proteins. The top ten most abundant proteins are listed. Among them, ENO1, which was previously reported to regulate the glycolysis drew our attention (Fig. 3A). ENO1 which is the key enzyme modulating the global levels of glycolysis through regulating the production of phosphoenolpyruvate (PEP), may be a potential substrate for SENP1 (Fig. 3B). Thus, we hypothesized that ENO1 is a candidate protein regulated by SENP1. The binding of SENP1 to ENO1 in MKN-45 and HGC-27 cells was confirmed by Co-IP (Fig. 3C and D). Additionally, the exogenous binding between Flag-SENP1 and HA-ENO1 was validated in HEK293T cells transfected with the respective constructs (Fig. 3E). Thus, we confirmed that ENO1 can bind to SENP1 in GC. To verify ENO1 SUMOylation in GC cells, we precipitated the ENO1 protein in GC cells, then we detected the SUMO in different samples. The results showed that ENO1 could be SUMOylated and SUMO2/3 was the primary SUMO type that binds to ENO1. Since SENP1 could deSUMOylate the target protein, we investigated whether SENP1 could mediate ENO1 SUMOylation and found that the SUMOylation of endogenous ENO1 increased after SENP1 knockdown (Fig. 3F). Supportively, Immunofluorescence analysis revealed that SENP1 colocalized with ENO1 in GC cells (Fig. 3G).

To map the major region of SENP1 that binds to ENO1, the different truncations were constructed [42], as shown in Fig. 3H. The results showed that the truncation that lack S1 region was incapable to bind with full length ENO1. Meanwhile, we constructed different domain of ENO1 on plasmid vector. Co-IP assay revealed that M2 of ENO1 could bind to SENP1 (Fig. 3I, J), M1 could partially bind to SENP1. Though Molecular-docking assay, we further verified the interaction between SENP1 and ENO1, and the binding domain of each protein was similar to above result (Fig. 3K). Thus, our strong evidence shows that SENP1 could directly bind with and deSUMOylate ENO1.

### SUMO2-modified ENO1 is desumoylated and stabilized by SENP1

**Fig. 4:**
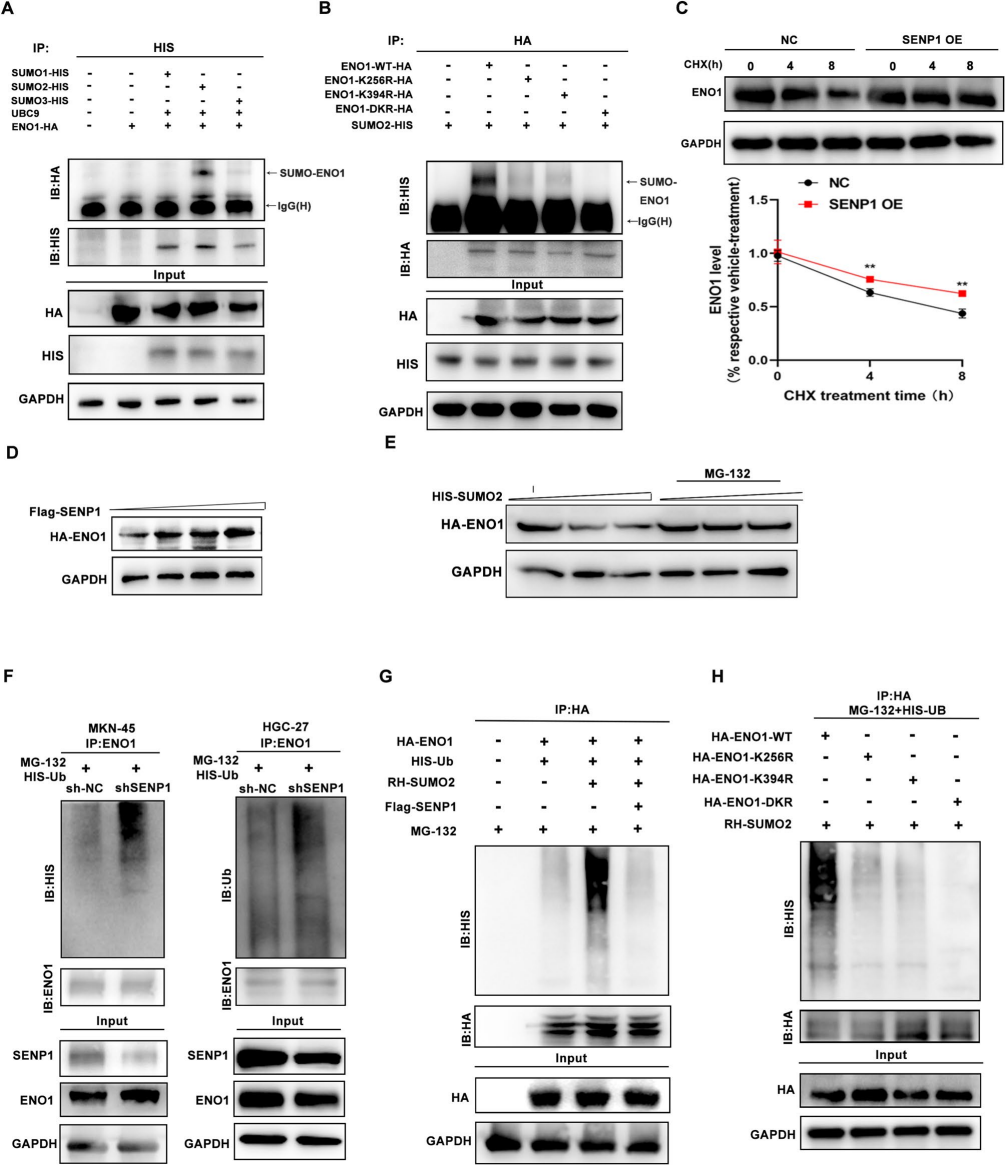
SUMO2-modified ENO1 is deSUMOylated and stabilized by SENP1. **A** SUMO1, SUMO2 and SUMO3 ligation with ENO1 protein in HEK293T cells transfected with ENO1-HA, UBC9, and HIS-SUMO1/HIS-SUMO2/HIS-SUMO3. **B** 293T cells were transfected with HA-tag ENO1, wild-type (WT), ENO1-K394R, ENO1-K256R, or ENO1-DKR with HIS-SUMO2. ENO1 SUMOylation was detected by immunoprecipitation with anti-HA antibody and then western blotting with anti-HIS antibodies. **C** Western blotting showing that the overexpression of SENP1 in HGC-27 cells resulted in decelerated degradation of ENO1. **D** 293T cells were transfected with HA-ENO1 and increasing amounts of Flag-SENP1 for 48 h. Lysates were prepared and analyzed by western blotting. **E** 293T cells were transfected with HA-ENO1 and increasing amounts of HIS-SUMO2 for 48 h. Cells were incubated with DMSO/MG-132 for 6 h. Lysates were prepared and analyzed by western blotting. **F** Co-IP showing that the knockdown of SENP1 increased accumulation of polyubiquitinated ENO1 in MKN-45 and HGC-27 cells. Anti-HIS antibody was used to bind HIS-tagged Ub to indicate ubiquitination. **G** 293T cells were transfected with Flag-SENP1, HIS-ubiquitin (HIS-Ub), RH-SUMO2 and HA-ENO1 and treated with MG132 (10 μm) for the last 10 h. The ubiquitination of HA-ENO1 was determined by immunoprecipitation (IP) assay using HA-beads and western blotting using antibody against HIS. **H** 293T cells with expression of RH-SUMO2, wild-type ENO1 or mutant ENO1 plasmids were transfected with HIS-Ub for 24 h. Cell lysates were subjected to Co-IP using an anti-HA antibody, and Western blot was performed using the indicated antibodies. *p < 0.05; **p < 0.01; ***p < 0.001

To explore the specific type of SUMO binding to ENO1, we transfected ENO1, HIS-SUMO1, HIS-SUMO2, HIS-SUMO3, and the UBC9 plasmid into HEK293T cells. Our results highlighted that ENO1 was predominantly SUMOylated by SUMO2 (Fig. 4A). We then sought to identify the potential SUMO sites in ENO1 by using SUMO plot, JASSA software, GPS-SUMO (http://sumosp.biocuckoo.org/index.php) analysis. Based on the overlapping predictions from three databases, the K256 and K394 residues of ENO1 were predicted to be the probable target sites that could bind to SUMO2. Then, plasmids encoding single-site mutant ENO1, in which the corresponding Lys (K) residue was replaced with Arg (R), were synthesized and transfected into HEK293T cells. The SUMOylation of both ENO1-K256R and ENO1-K394R was notably reduced compared to wild-type ENO1. Furthermore, the SUMOylation of the double mutant form (DKR) of ENO1 was completely abolished (Fig. 4B).

Growing evidence indicates that the SUMOylation status of a substrate protein affects its stability, subcellular distribution, special function, and ability to bind with other proteins [43]. Thus, we first investigated the effect of SUMOylation on ENO1 stability. We found that SENP1 knockdown suppressed ENO1 expression in GC cells. As expected, the opposite outcome was observed in the SENP1 overexpression group (Supplementary Fig. 5A-B). We speculated that the reduced expression of ENO1 due to SENP1 knockdown might be linked to alterations in ENO1’s stability. To test this, we utilized cycloheximide, a protein synthesis inhibitor, in GC cells. Our data suggested that SENP1 overexpression enhanced the stability of ENO1 (Fig. 4C). The protein level of ENO1 gradually increased when more SENP1 plasmid was transfected into cells (Fig. 4D). In addition, the protein level of ENO1 was dose-dependently decreased by HIS-SUMO2 overexpression, but the effect of SUMO2 on ENO1 expression was abolished by treatment with MG132 (Fig. 4E). MG132 inhibits proteasome activity, thereby we supposed that SUMOylated ENO1 protein are degraded via the proteasome pathway. Therefore, we postulated that SENP1 may regulate ENO1 protein level at posttranslational modification.

In support of this speculation, the results of ubiquitination assays demonstrated that knockdown of SENP1 dramatically increased the polyubiquitination of ENO1 (Fig. 4F). Co-expression of HA-ENO1, RH-SUMO2, HIS-Ub, and Flag-SENP1 in HEK293T cells revealed that SENP1 decreased the ubiquitination of ENO1 induced by SUMO2 overexpression (Fig. 4G). Ubiquitination assays were performed with the ENO1 mutants, and the reduction of ubiquitination was observed in both ENO1-K256R and ENO1-K394R mutants, and ENO1 had the most reduction of ubiquitination in DKR mutant (Fig. 4H). Therefore, these data indicate that SENP1 deubiquitinates and stabilizes ENO1 proteins.

To determine other functional consequences of ENO1 SUMOylation regulated by SENP1, we measured subcellular distribution of ENO1 in GC cells after SENP1 knockdown. Subcellular distribution of ENO1 did not change after SENP1 knockdown (Supplementary Fig. 5C). We expressed wild-type or different mutant forms of ENO1 in GC cells and measured subcellular distribution of ENO1-HA, which was unaffected (Supplementary Fig. 5D). We next measured enolase activity in GC cells and found that knockdown of SENP1 did not affect enolase activity of GC cells (Supplementary Fig. 5E). Then we stably expressed wild-type or double mutant forms (DKR) of ENO1 in GC cells, replacing endogenous ENO1 by knockdown with shRNA. Knockdown of ENO1 led to a significant reduction in enolase activity compared to that in the control, whereas DKR mutation ENO1 did not affect kinase activity (Supplementary Fig. 5F).

In conclusion, SENP1 deSUMOylates ENO1 via SUMO sites K256 and K394, and stabilizes ENO1 protein.

### SENP1 promotes GC progression depending on ENO1

**Fig. 5:**
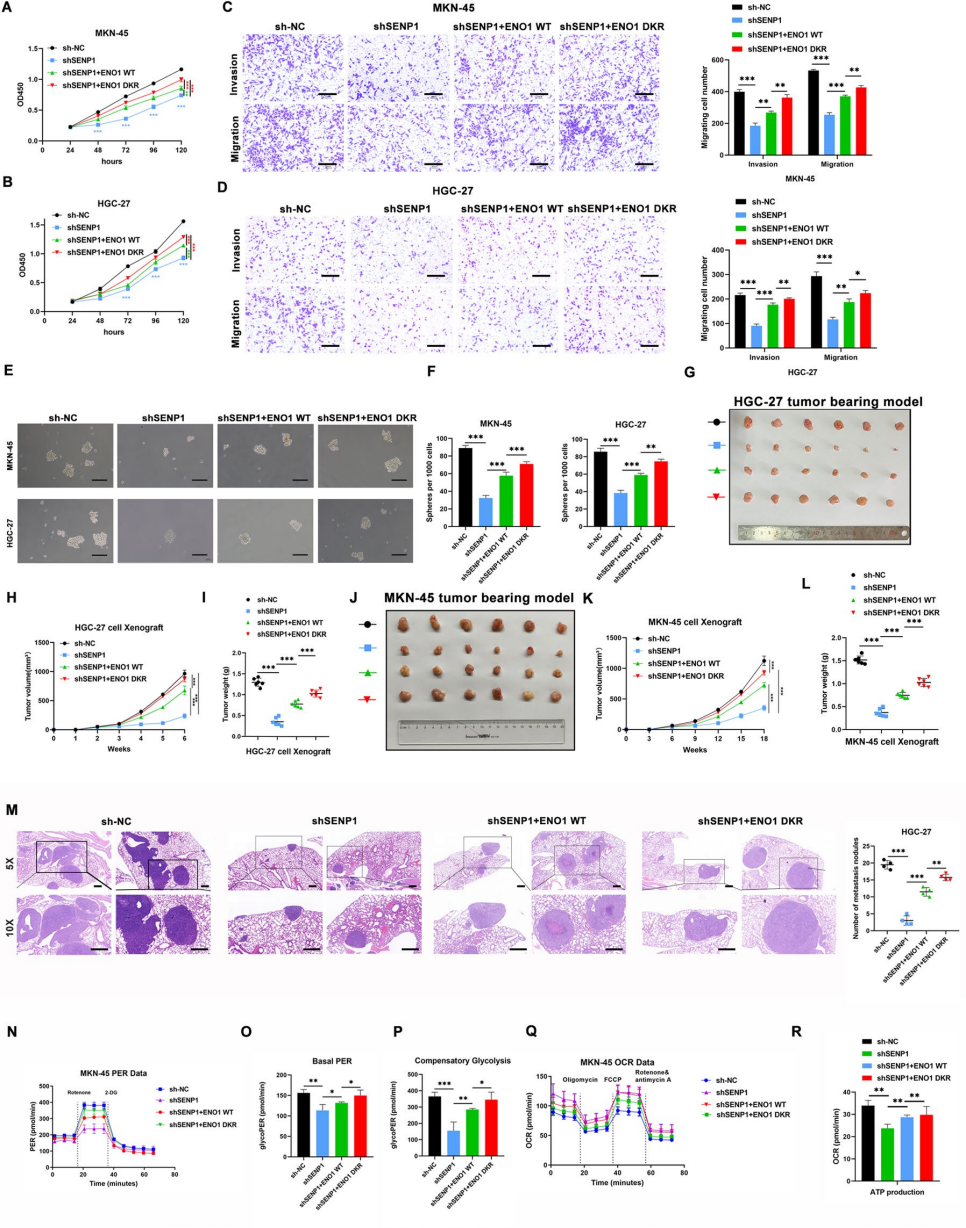
SENP1 promotes GC progression depending on ENO1. **A-B** CCK-8 assay of MKN-45, HGC-27 cells transfected as indicated. **C-D** Cell invasion and migration assays of GC cells transfected as indicated. Scale bar, 500 μm. **E-F** Representative images of formatted spheres among indicated cells. Scale bar, 100 μm. **G** The images of dissected tumors from HGC-27 cells stably transfected as indicated. **H-I** Tumor weights and sizes are represented as means of tumor weights (**I**)/sizes (**H**) ± standard deviation (SD). **J** The images of dissected tumors from HGC-27 cells stably transfected as indicated. **K-L** Tumor weights and sizes are represented as means of tumor weights (**L**)/sizes (**K**) ± standard deviation (SD). **M** Representative images of HE staining of lung tissues from HGC-27 cells stably transfected as indicated. **N-P** MKN-45 cells as described cells were measured by the Glycolytic Rate Assay Kit to determine glycolytic proton efflux rate (PER). Data are presented as mean ± SEM of three biologically independent samples. **Q-R** MKN-45 cells as described cells were measured by the mitochondrial stress kit to determine oxygen consumption rate (OCR). Data are presented as mean ± SEM of three biologically independent samples. Scale bar, 500 μm. *p < 0.05; **p < 0.01; ***p < 0.001

We hypothesized that SENP1 promotes GC cell malignancy by deSUMOylating and stabilizing ENO1. To verify this hypothesis, we introduced both wild-type and double-mutant ENO1 into GC cells. ENO1 overexpression effectively reversed the proliferation and migration inhibition induced by SENP1 knockdown in GC cells. Furthermore, the ENO1 mutants enhanced this effect (Fig. 5A-D). Spheroid experiments highlighted that overexpressing either wild-type or mutant ENO1 rescued the compromised stemness resulting from SENP1 knockdown (Fig. 5E-F).

To delve deeper into ENO1’s role in SENP1-mediated proliferation and migration in GC, we established xenograft subcutaneous tumor models and tail vein lung metastasis models. The results demonstrated that ENO1 overexpression restored the reduced proliferation and migration abilities in GC cells due to SENP1 knockdown, with the double mutation exhibiting even more pronounced effects (Fig. 5G-M).

As ENO1 is a key regulator in the Warburg effect, its effect on SENP1 in glycolysis was investigated. ENO1 overexpression alleviated the weakened glycolytic capacity caused by SENP1 knockdown, with the double mutation exhibiting even more pronounced effects (Fig. 5N-R).

On the contrary, silencing ENO1 suppressed cell proliferation induced by SENP1 (Supplementary Fig. 6A-B), as well as cell invasion and migration (Supplementary Fig. 6C-D). Similar results were shown in xenograft subcutaneous tumor models (Supplementary Fig. 6E-J).

In summary, our investigation reveals the dependency of SENP1’s impact on the malignant progression of GC on ENO1.

### SUMO-defective ENO1 contributes to GC cell proliferation and tumorigenesis

To explore whether SUMOylation of ENO1 modulated the cancer cell aggressive phenotype, we performed CCK8 assays, Transwell assays and Spheroid experiments to detect malignant phenotype with different ENO1 mutations. While knockdown of endogenous ENO1 markedly reduced cell proliferation, ectopic expression of wild-type rescued the effect of ENO1 silencing. DKR mutant ENO1 even accelerated cell proliferation compared with wild-type (Supplementary Fig. 7A). Similar results were witnessed in Transwell assays and Spheroid experiments (Supplementary Fig. 7B-E). Meanwhile, wild-type ENO1 overexpression alleviated the weakened glycolytic capacity caused by ENO1 knockdown, which was further enhanced in DKR mutant ENO1 (Supplementary Fig. 7F, 7G). To test tumorigenic potential in vivo, HGC-27 and MKN-45 cells were used in subcutaneous tumorigenesis assays. Consistently, Wild-type ENO1 restored decreased tumor burden caused by ENO1 knockdown, and DKR mutant ENO1 promoted tumorigenesis more than wild-type ENO1 (Supplementary Fig. 7H-M). These results demonstrate that SUMO-defective ENO1 might contribute to GC cell proliferation and migration.

### Momordin Ic suppressed the GC cell growth

It is conceivable that SENP1 inhibitor Momordin Ιc (Mc) has negative effect to GC cell growth. To further confirm the role of Mc in GC cells, we detected the viability of cells under Mc treatment. The half-maximal inhibitory concentrations (IC50) for Mc in MKN-45 and HGC-27 cell lines were determined to be 16.73 µM and 14 µM respectively (Supplementary Fig. 8A, 8B). The expression of SENP1 and ENO1 was significantly decreased after Mc treatment (Supplementary Fig. 8C). And Mc treatment enhanced ENO1 SUMOylation in GC cells (Supplementary Fig. 8D).

Next, the anti-tumor effects of Mc were determined in vivo using a CDX mouse model. Mc markedly suppressed the tumor growth (Supplementary Fig. 8E-G). Consistent with an inhibition of SENP1 activity, Mc treatment also led to the accumulation of SUMO2/3-modified proteins in HGC-27 tumor xenografts (Supplementary Fig. 8H). These data suggest that Mc significantly inhibits GC progression by targeting SENP1.

### Momordin Ic enhances the anti-tumor effects of cisplatin

**Fig. 6:**
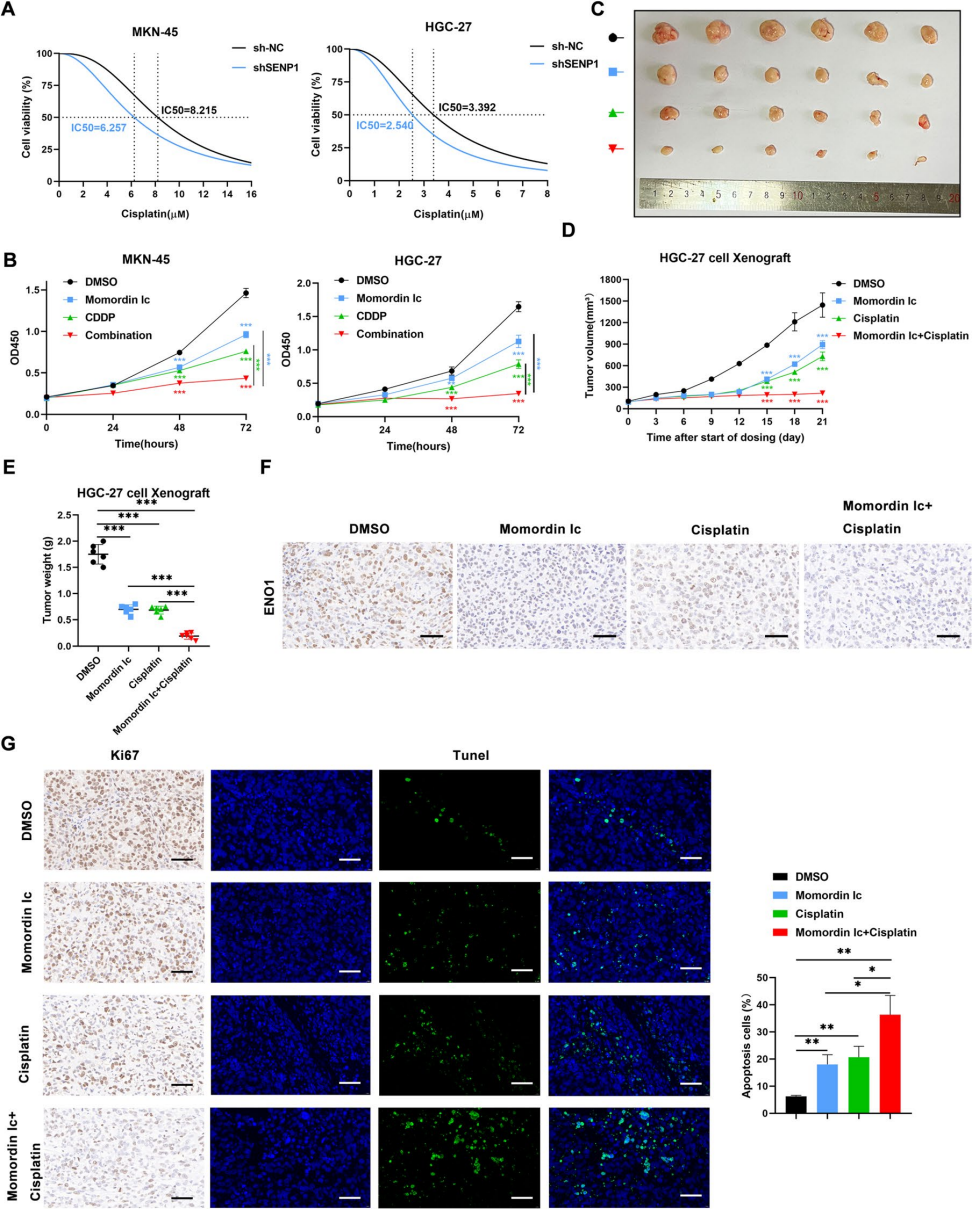
Momordin Ic enhances the anti-tumor effects of cisplatin. **A** The effect of SENP1 on the chemosensibility of GC cells was measured by CCK8 assay after 72 h treated with cisplatin. Each bar represents mean ± SD. **B** GC cell growth was compared by CCK8 treated with treated with Momordin Ic alone (16.7 µM in MKN-45 cells and 14 µM in HGC-27 cells), cisplatin alone (8.2µM in MKN-45 cells and 3.4 µM in HGC-27 cells), and combination. **C-E** Tumor growth was compared between xenograft nude mice injected with tumor treated with Momordin Ic alone (10 mg/kg), cisplatin alone (5 mg/kg), and combination (*n* = 6) every 4 days. **F** Representative IHC staining of ENO1 from tumors in (**C**). Scale bars, 50 μm. **G** Ki67 and Tunel were analyzed in a representative CDX tumor treated with treated with Momordin Ic alone (10 mg/kg), cisplatin alone (5 mg/kg), and combination by IHC and mIHC. Scale bar, 50 μm. *p < 0.05; **p < 0.01; ***p < 0.001

Cisplatin is first-line treatment for gastric cancer. It has been reported that SENP1 and SUMOylation is correlated to chemotherapy sensitivity of tumors [8, 44–48]. SENP1 can reduce cisplatin resistance and increase the sensitivity of different cancer cells to cisplatin [8, 44–48]. To further investigate the therapeutic benefit of targeting SENP1 in combination with cisplatin, the sensitivity of cisplatin in SENP1 knockdown cells was tested. It was found that endogenous SENP1 knockdown significantly enhanced the sensitivity to cisplatin treatment (Fig. 6A). The combination of cisplatin and Mc exhibited a more potent inhibitory effect on the growth of GC cells compared to cisplatin alone (Fig. 6B). A xenograft model of HGC-27 cells in nude mice was established to analyze the combination effect of cisplatin and Mc in vivo. The combination of cisplatin and Mc showed strong inhibition of GC cells growth, which was much higher than that of cisplatin or Mc alone (Fig. 6C-E). Furthermore, reduced ENO1 expression was observed in the tumors of cisplatin + Mc treated group (Fig. 6F). The level of Ki67 was significantly suppressed in the combination treatment group. And GC cell apoptosis was detected by TUNEL Apoptosis Detection Kit with fluorescence microscope. And combination treatment group increased the apoptosis radio of GC cells (Fig. 6G). In summary, these data suggest that targeting SENP1 significantly enhances the anti-tumor effects of cisplatin on gastric cancer xenografts.

### Upregulation of the SENP1-ENO1 axis is associated with poor outcomes in GC patients

**Fig. 7:**
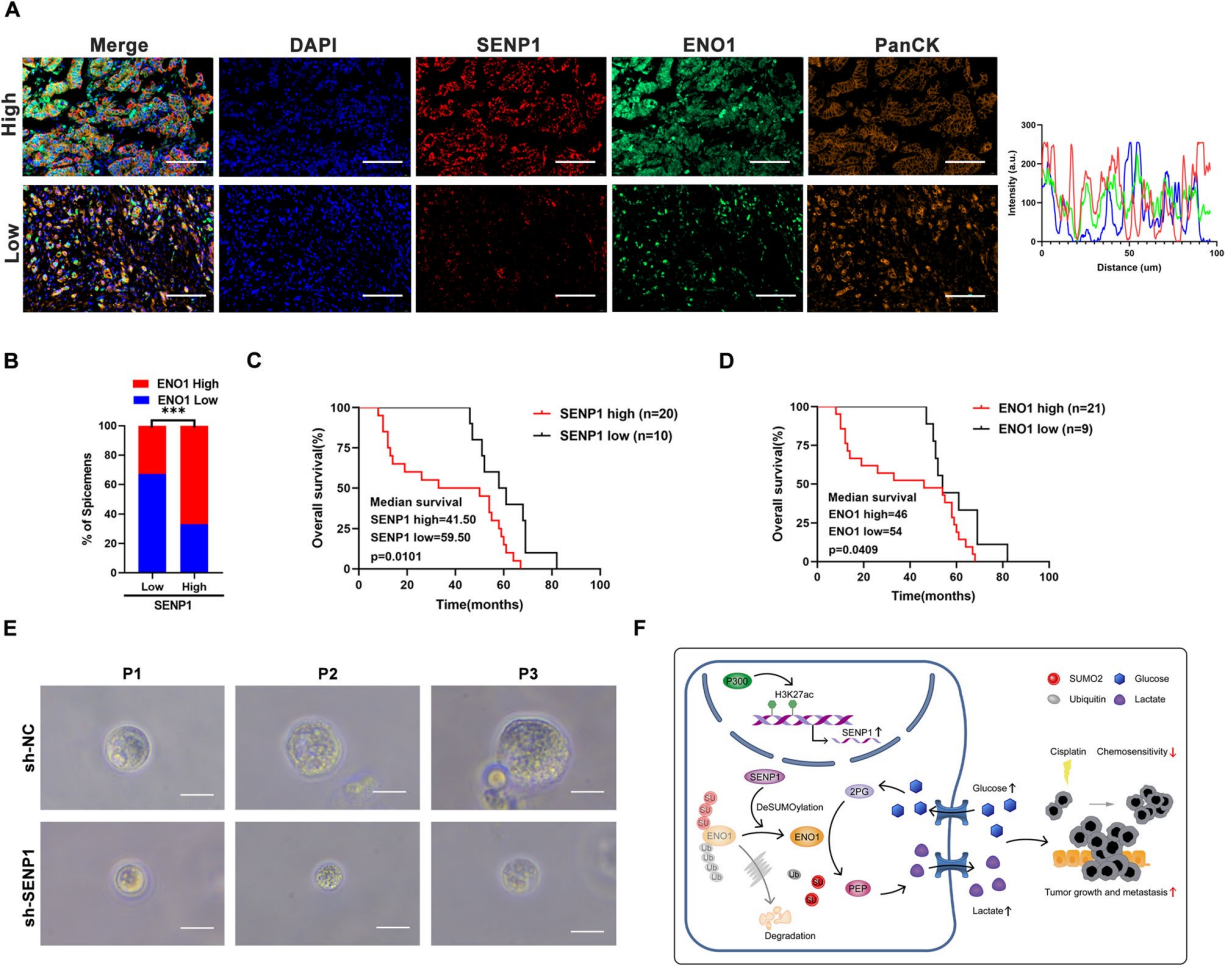
Upregulation of the SENP1-ENO1 axis is associated with poor outcomes in GC patients. **A** Representative image of FISH assay of SENP1and ENO1 in 30 clinical GC samples. Nuclei were stained with DAPI. Cancer cells were identified with PanCK. Scale bars: 100 μm. **B** Correlation analysis of SENP1 and ENO1 expression in (A); chi-square test. **C-D** Kaplan–Meier plots of overall survival of 30 GC patients stratified by protein expression of SENP1 (**C**) and ENO1 (**D**) showing that SENP1 high and ENO1 high GC patients displayed significantly shorter overall survival than SENP1 low and ENO1 low GC patients. **E** Effects of SENP1 knockdown on the growth of gastric organoids. Scale bar: 50 μm. **F** Schematic diagram showing how SENP1 regulated ENO1 levels in tumor cells and promoted GC progression. *p < 0.05; **p < 0.01; ***p < 0.001

To explore the clinical implications of the SENP1/ENO1 axis, we assessed the expression of SENP1 and ENO1 in serial sections of 30 human GC specimens via multiplex immunohistochemical (mIHC) assay. The analysis revealed a positive correlation between the expression levels of SENP1 and ENO1 (Fig. 7A, B). Furthermore, survival analysis showed that high SENP1 expression and high ENO1 expression were positively associated with unfavorable outcomes for GC patients (Fig. 7C, D). We also established a GC organoid model generated from different patients with GC to further verify the potential clinical value of SENP1. The results showed that overexpression of SENP1 by lentivirus significantly promoted GC cell proliferation in the GC organoids, while knockdown of SENP1 decreased GC cell proliferation (Fig. 7E).

## Discussion

SUMOylation is a common post-translational modification during various cellular processes, involving cell metabolism, RNA metabolism, cell cycle progression, DNA damage response and so on [43, 49]. Growing evidence showed that SUMOylation is involved in cancer pathogenesis, and some studies of drugs targeting the SUMO modification process (such as TAK-981 and Momordin Ic) have made great progress [8, 50–52]. The SUMOylation status of substrate proteins is dynamic, which regulated by the family of SENPs. Many previous studies have highlighted the pivotal role of SENPs in the development of cancer [11, 53]. However, the specific role of SENPs in GC progression remain unclear. In this study, we confirmed that SENP1 expression was increased in GC and associated with poor prognosis. Furthermore, we firstly unveiled that SENP1 promoted GC cell proliferation and invasion by using *in vivo and in vitro* experiment. Previous studies have reported that SENP1 could affect the cancer stemness of HCC or CRC cells [11, 54]. Therein, we also determined that whether the stemness of GC cells was influenced by ectopic SENP1 expression. Accordingly, our data implied that SENP1 could maintain the stem cell-like characteristics of GC cells. Thus, SENP1 is a candidate oncogene and a potential biomarker in GC. Previous studies have shown that SENP1 and SUMOylation are closely associated with glycolysis, which is one of the most important malignant phenotypes of tumors [20, 21, 40, 41]. For instance, a recent study has reported that deSUMOylated hexokinase 2 (HK2) influences mitochondria function in prostate cancer cells [20]. To validate the correlations of SENP1 with glycolysis, the gene set GSE183904 was selected, including a total of 31 cases of primary GC and 9 normal tissues, for single-cell sequencing result annotation. In gastric cancer, high expression of SENP1 is positively correlated with glycolysis activation. Consistently, our metabolic flux analysis showed that SENP1 promotes GC cell to favor glycolysis for energy production, rather than the tricarboxylic acid (TCA) cycle.

To uncover the underlying glycolytic mechanism of SENP1 function in GC development, mass spectrometry analysis was conducted and proteomics analysis. Among SENP1 binding proteins, we assumed that ENO1 may be a potential candidate substrate protein of SENP1 in GC cells because of its high binding abundance. ENO1 is a well-known glycolytic enzyme which ubiquitously expressed in most mammalian cells, the function of ENO1 is to hydrolyze 2-phosphoglycerate (2-PG) to phosphoenolpyruvate (PEP), which ultimately generate ATP to sustain the malignant activity of cancer cells [55, 56]. Previous study has shown that ENO1 promoted non-small cell lung cancer proliferation and metastasis through regulating glycolysis, cell cycle and epithelial-mesenchymal transition (EMT)-associated genes [57]. Importantly, ENO1 enhanced the proliferation and invasion in GC cells through ENO1/AKT axis [28]. Thus, we selected the ENO1 as a candidate substrate protein that modulated by SENP1.

Next, we investigated whether ENO1 is a SUMO substrate. In the light of our results, we found that ENO1 is primarily modified by SUMO2, and its de-SUMOylation is regulated by SENP1. We further confirmed that SENP1 regulate the deSUMOylation of ENO1 at residues K256 and K394. Post-translational modifications (PTMs) of ENO1 are the important mechanism for regulating protein function and can alter the catalytic activity, localization and protein stability of ENO1 [58, 59]. And SUMOylation triggered ubiquitination of ENO1 [60]. Thus, the status of SUMOylation can affect the stability of protein itself, the enzyme activity, subcellular location, and interaction of other protein [43]. The function of ENO1 depends on its localization. The cytoplasm is the major expression site of ENO1, where ENO1 catalyzes glycolysis, maintains mitochondrial membrane stability, regulates signaling pathways, and reorganizes the cytoskeleton [61–63]. While in the nucleus, MBP1 (c-MYC promoter binding protein), the alternative translation variant of ENO1, suppresses c-MYC and promotes the transcriptional level of ENO1 mRNA [27]. Our evidence indicated that SENP1-mediated deSUMOylation could stabilize ENO1 through decreasing proteasome-dependent ubiquitination degradation. Moreover, we also found that SENP1 has no effect on the nuclear entry and enolase activity of ENO1 in GC cells.

According to our study, we assumed that SENP1 is an oncogenic gene in the progression of GC. Consequently, SENP1 inhibition may be a promising therapeutic strategy for patients with GC. Momordin Ic (Mc), a natural SENP1 inhibitor, has been identified to directly bind and inhibit SENP1 in multiple myeloma, prostatic cancer and ovarian cancer [64–66]. We discovered that tumor burdens were noticeably extenuated in the Mc treatment groups in CDX mouse model. Given the significance of platinum-containing regimens in the first-line treatment of GC and the inevitable platinum-resistance, whether Mc could enhance the anti-tumor effects of cisplatin on GC was further investigated. Our results powerfully proved that Mc has a therapeutic effect on GC and can enhance the sensitivity of GC cells to cisplatin. Our study provides rationale for further exploration of SENP1 inhibition for clinical application for solid malignancies.

## Conclusion

Our study indicates that SENP1 acts as an oncogene in GC and emphasize the important role of SENP1/ENO1 axis in cancer glucose metabolism reprogramming. These findings contribute to the selection of therapeutic modalities in clinical practice and propose a strategy to target SENP1 as a potential adjuvant therapy in combination with Cisplatin for GC treatment.

## Supplementary Information

Below is the link to the electronic supplem

entary material.

Supplementary Material 1

Supplementary Material 2

Supplementary Material 3

## Data Availability

All data in our study are available upon request.

## Abbreviations

GC: Gastric cancer
STAD: Stomach adenocarcinoma
DMSO: Dimethyl sulfoxide
CCK8: Cell counting kit-8
IHC: Immunohistochemistry
CHX: Cycloheximide
OS: Overall Survival
PBS: Phosphate buffer saline
DFS: Disease-free-survival
PMSF: Phenylmethylsulfonyl fluoride
qRT-PCR: Quantitative Reverse Transcription PCR
Co-IP: Co-Immunoprecipitation
CHIP: Chromatin Immunoprecipitation
SUMO: Small ubiquitin-like modifier
SENP1: Sentrin-specific protease1
ENO1: Alpha-Enolase
