# Supplementary material for: SENP1 drives glycolysis and cisplatin resistance in gastric cancer via desumoylating ENO1
Source: J Exp Clin Cancer Res. 2025 Oct 8;44:285. doi: 10.1186/s13046-025-03543-z (PMC12506269; doi:10.1186/s13046-025-03543-z)
Supplement: Supplementary file 1 — Supplementary Material 1 [file 13046_2025_3543_MOESM1_ESM.docx]

**Supplementary Materials for**

­ **SENP1 drives glycolysis and cisplatin resistance in gastric cancer via deSUMOylating ENO1**

Yuan Fang ^a,1^, Yunru Gu ^a,1^, Tingting Xu ^a,1^, Peng Wang ^b, 1^, Xi Wu ^c^, Haoyang Shen ^a^, Yangyue Xu ^a^, Zixiang Xu ^a^, Lei Cao ^d^, Xiao Li ^e, *^, Hao Wu ^a, *^, Yongqian Shu ^a, f, *^, Pei Ma ^a, d, f, g *^


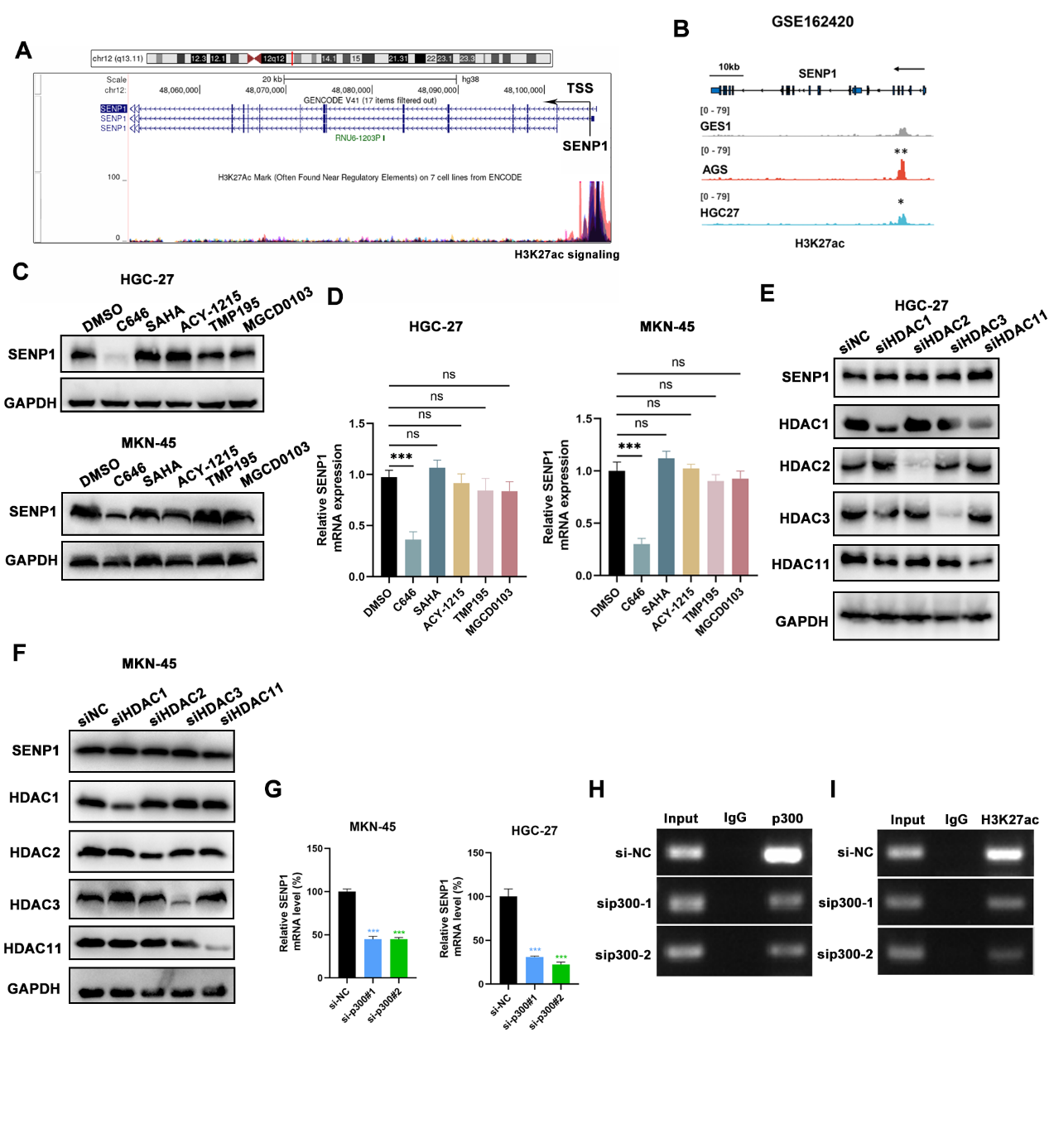


**Fig. S1 p300-mediated H3K27ac activates SENP1 transcription in GC.**

**A-B** Data from the UCSC genome bioinformatics site (<http://genome.ucsc.edu/>) and GSE162420 showed high enrichment of H3K27ac in the promoter of SENP1. **C-D** Relative (**C**) protein and (**D**) mRNA levels of SENP1 in HGC-27 and MKN-45 cells treated with C646 and different HDAC inhibitors. **E–F** Relative protein levels of the SENP1 and HDAC in HGC-27 and MKN-45 cell lines with or without knockdown of HDAC1, HDAC2, HDAC3 or HDAC11. **G** Relative mRNA levels of SENP1 in HGC-27 and MKN-45 cell lines with or without knockdown of p300. **H-I** ChIP assays were used to determine the level of p300 binding (H) and the enrichment of H3K27ac (I) at the promoter of SENP1 in p300 deficiency or control HGC-27 cells using agarose gel electrophoresis. The data are the means±SEMs of three independent experiments. *p <0.05; **p < 0.01; ***p < 0.001.


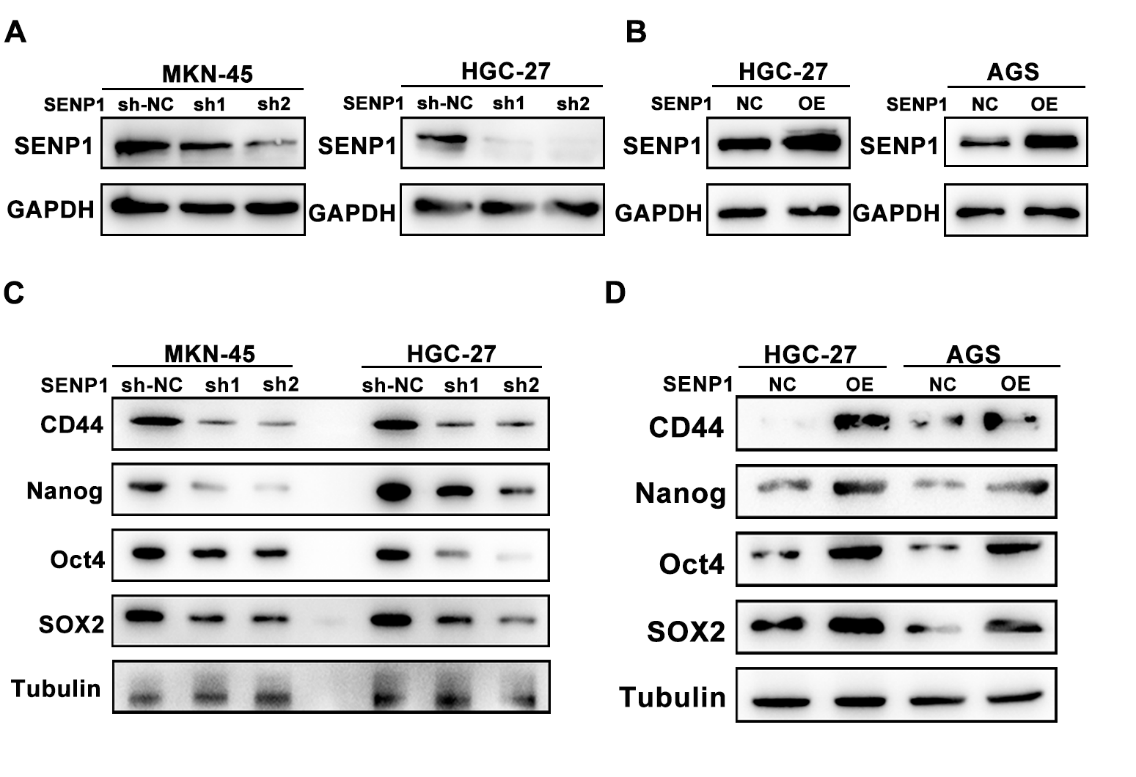


**Fig. S2 Stemness-related factors are upregulated by SENP1 in GC cells.**

**A** Western blotting assay of SENP1 expression in MKN-45 and HGC-27 cells transfected with shSENP1 or sh-NC control. **B** Western blotting assay of SENP1 expression in AGS and HGC-27 cells transfected with SENP1 OE or NC control. **C-D** Western blotting of stemness-related factors including CD44, SOX-2, OCT-4, and Nanog among indicated cells.


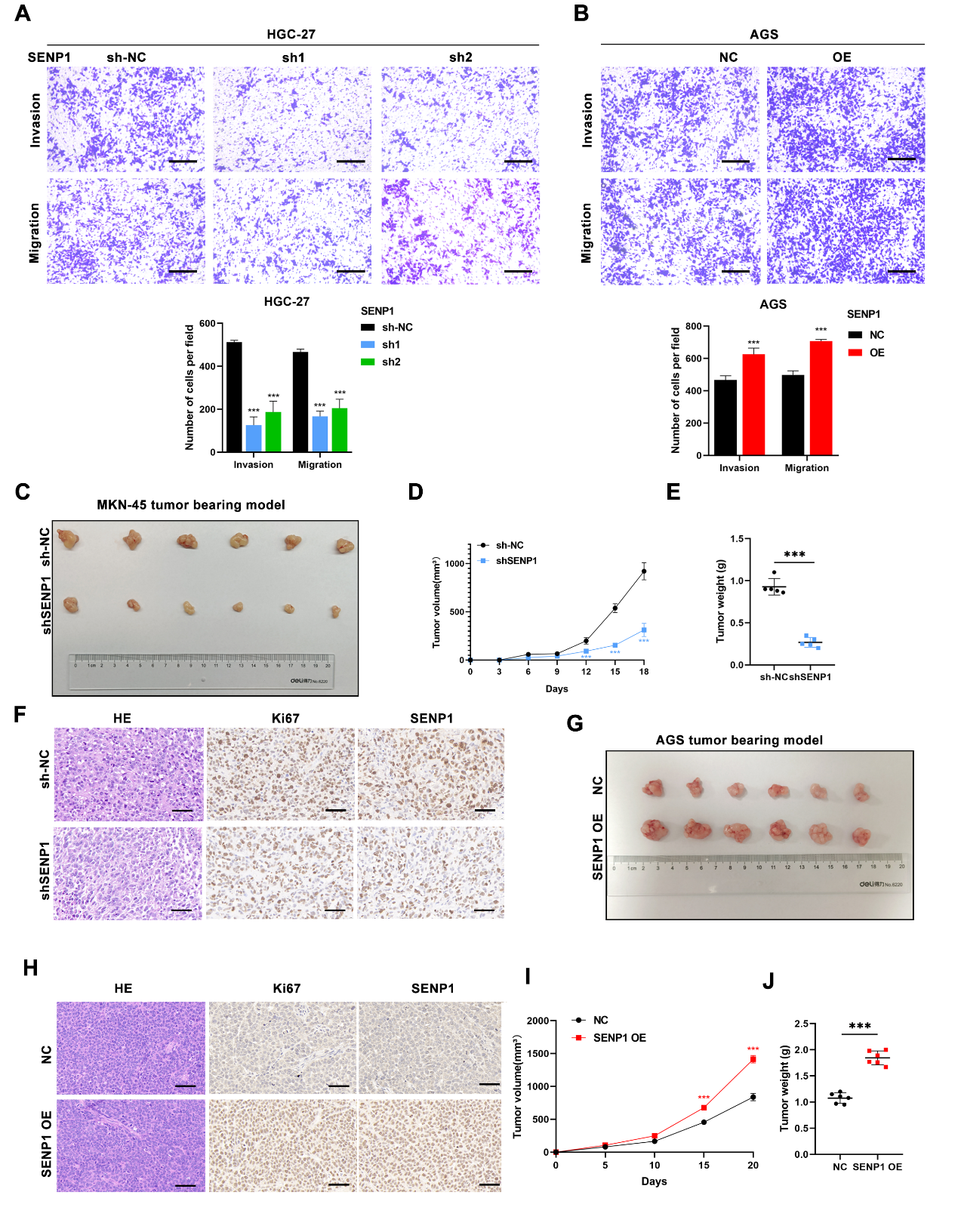


**Fig. S3 SENP1 promotes gastric cancer cell growth *in vivo***

**A-B** Cell invasion and migration assays of GC cells transfected as indicated. Scale bar, 500μm. **C** The images of dissected tumors from MKN-45 cells stably transfected with sh-NC and shSENP1. **D-E** Tumor weights and sizes are represented as means of tumor weights (E)/sizes (D) ± standard deviation (SD). **F** Tumor tissue samples were immunostained for haematoxylin and eosin (H&E), Ki‐67 and SENP1.Scale bar, 50μm. **G** The images of dissected tumors from AGS cells stably transfected with NC and SENP1 OE. **H** Tumor tissue samples were immunostained for haematoxylin and eosin (H&E), Ki‐67 and SENP1.Scale bar, 50μm.**I-J** Tumor weights and sizes are represented as means of tumor weights (J)/sizes (I) ± standard deviation (SD).


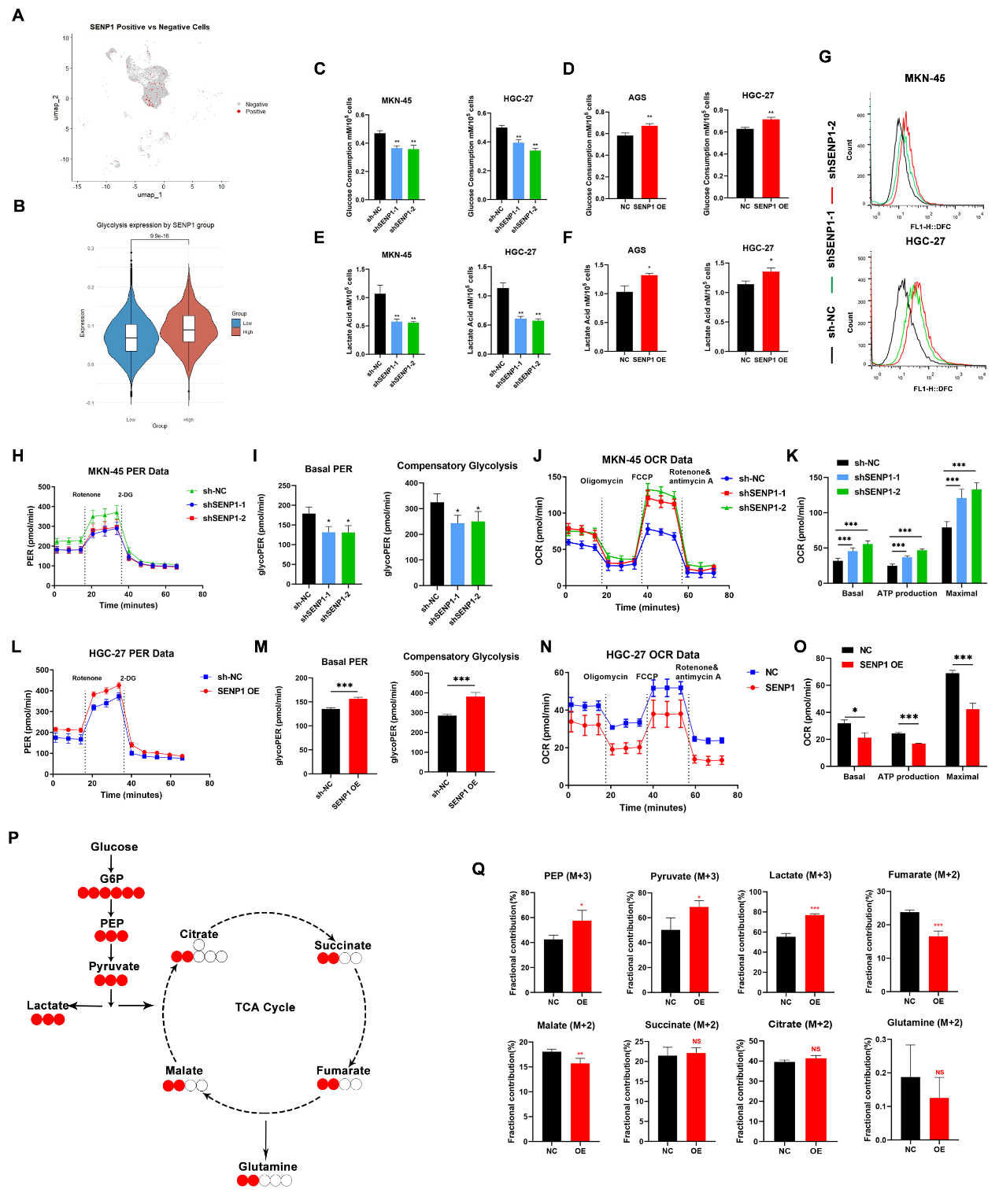


**Fig. S4 SENP1 regulates GC cells’ glycolysis partially through ENO1.**

**A-B** The gene set GSE183904 was selected for single-cell sequencing result annotation. High expression of SENP1 was positively correlated with glycolysis activation. **C–G** SENP1 knockdown and control vector cells were tested for glucose consumption (C), lactate production (E) and ROS level (G). SENP1 overexpression and control vector cells were tested for glucose consumption (D) and lactate production (F). **H-J** MKN-45 cells as described cells were measured by the Glycolytic Rate Assay Kit to determine glycolytic proton efflux rate (PER). Data are presented as mean± SEM of three biologically independent samples. **J-K** MKN-45 cells as described cells were measured by the mitochondrial stress kit to determine oxygen consumption rate (OCR). Data are presented as mean± SEM of three biologically independent samples. **L-M** HGC-27 cells as described cells were measured by the Glycolytic Rate Assay Kit to determine glycolytic proton efflux rate (PER). Data are presented as mean± SEM of three biologically independent samples. **N-O** HGC-27 cells as described cells were measured by the mitochondrial stress kit to determine oxygen consumption rate (OCR). Data are presented as mean± SEM of three biologically independent samples. **P** Schematic of glycolysis pathway flux and TCA pathway flux, illustrating labeling from [U-^13^C] glucose. **Q** Fractional labeling of metabolites of glycolysis pathway flux and TCA pathway flux in SENP1 overexpression and control vector cells cultured in medium containing [U-^13^C]-glucose for 15 min. Data represent mean values ± SD from three independent experiments *p <0.05; **p < 0.01; ***p < 0.001.


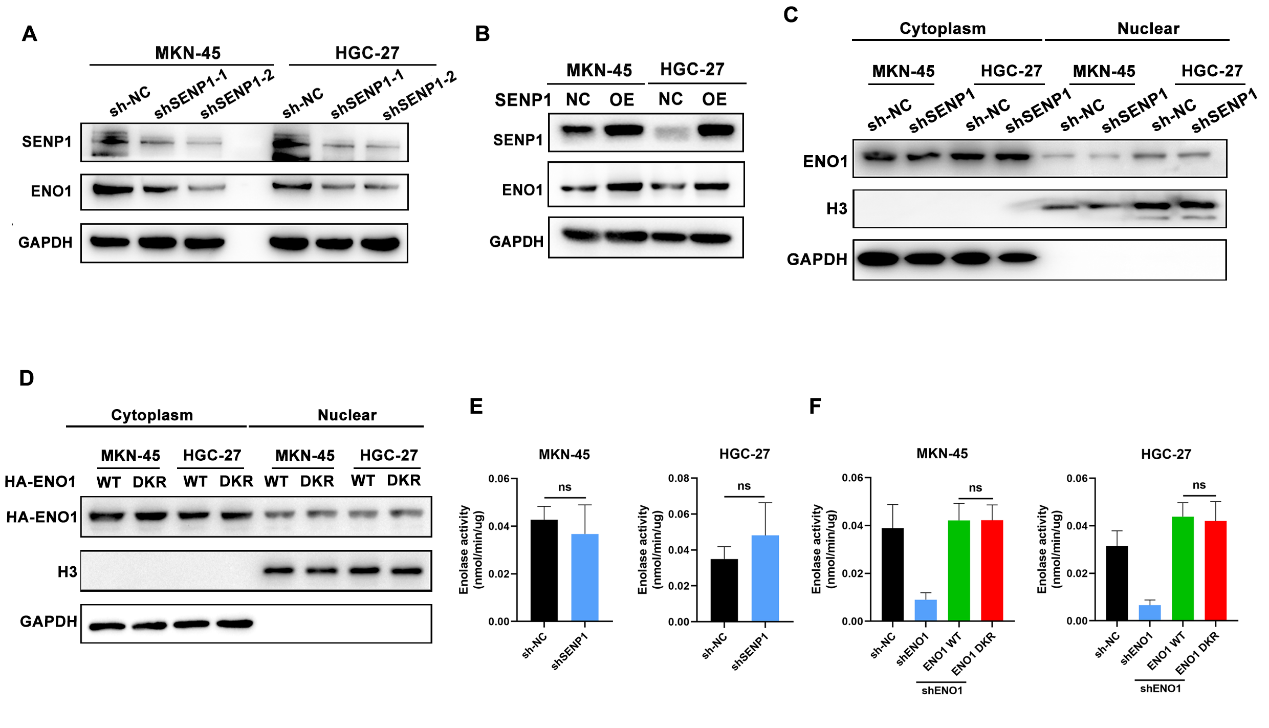


**Fig. S5 SENP1 does not affect subcellular distribution and enolase activity of ENO1 via de-SUMOylation.**

**A** Protein levels of ENO1 were downregulated after SENP1 deficiency, which determined by western blotting. **B** The protein levels of ENO1 in GC cells were measured by western blotting after SENP1 overexpression. **C** Western blotting showed endogenous ENO1 expression in nucleic and cytoplasmic extraction from SENP1 shRNA or sh-NC GC cells. **D** GC cells expressing different mutant forms of ENO1 were prepared for nucleic and cytoplasmic extraction and measured using HA and H3 antibodies. **E** Enolase activities were measured after SENP1 knockdown. **F** Enolase activities were measured after GC cells expressing different mutant forms of ENO1. *p <0.05; **p < 0.01; ***p < 0.001.


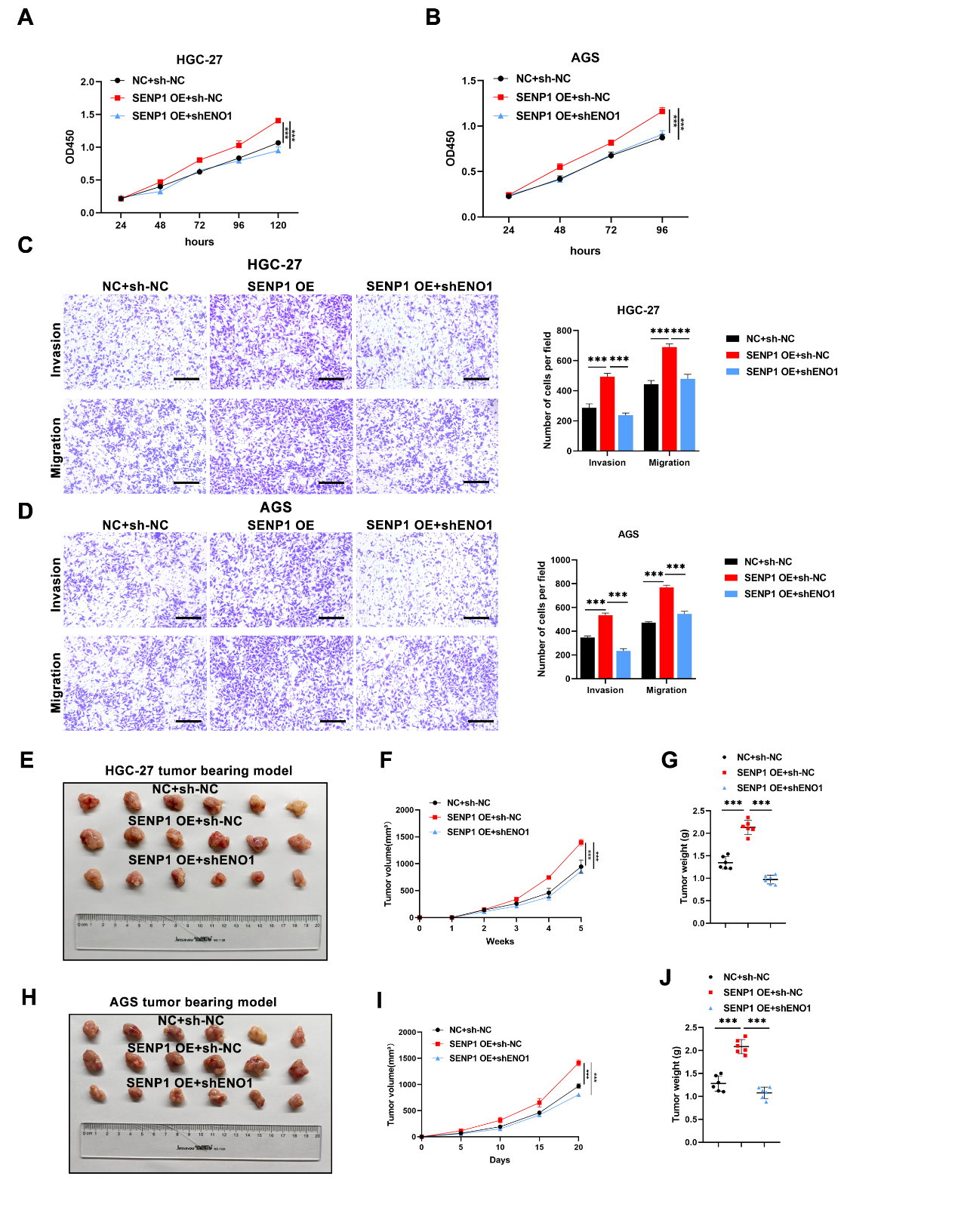


**Fig. S6. SENP1 promotes the GC progression by upregulating ENO1.**

**A-B** CCK8 assays were conducted to determine cell proliferation of SENP1-overexpressing HGC-27 and AGS cells transfected with the ENO1 shRNAs or their corresponding controls. **C-D** Transwell assays were conducted to determine cell migration and invasion abilities of SENP1-overexpressing HGC-27 and AGS cells transfected with the ENO1 shRNAs or their corresponding controls. **E** The images of dissected tumors from HGC-27 cells stably transfected as indicated. **F-G** Tumor weights and sizes are represented as means of tumor weights (G)/sizes (F) ± standard deviation (SD). (Scale bars, 100 mm) **H** The images of dissected tumors from AGS cells stably transfected as indicated. **I-J** Tumor weights and sizes are represented as means of tumor weights (J)/sizes (I) ± standard deviation (SD). (Scale bars, 100 mm) *p<0.05; **P< 0.01, *** p<0.001.


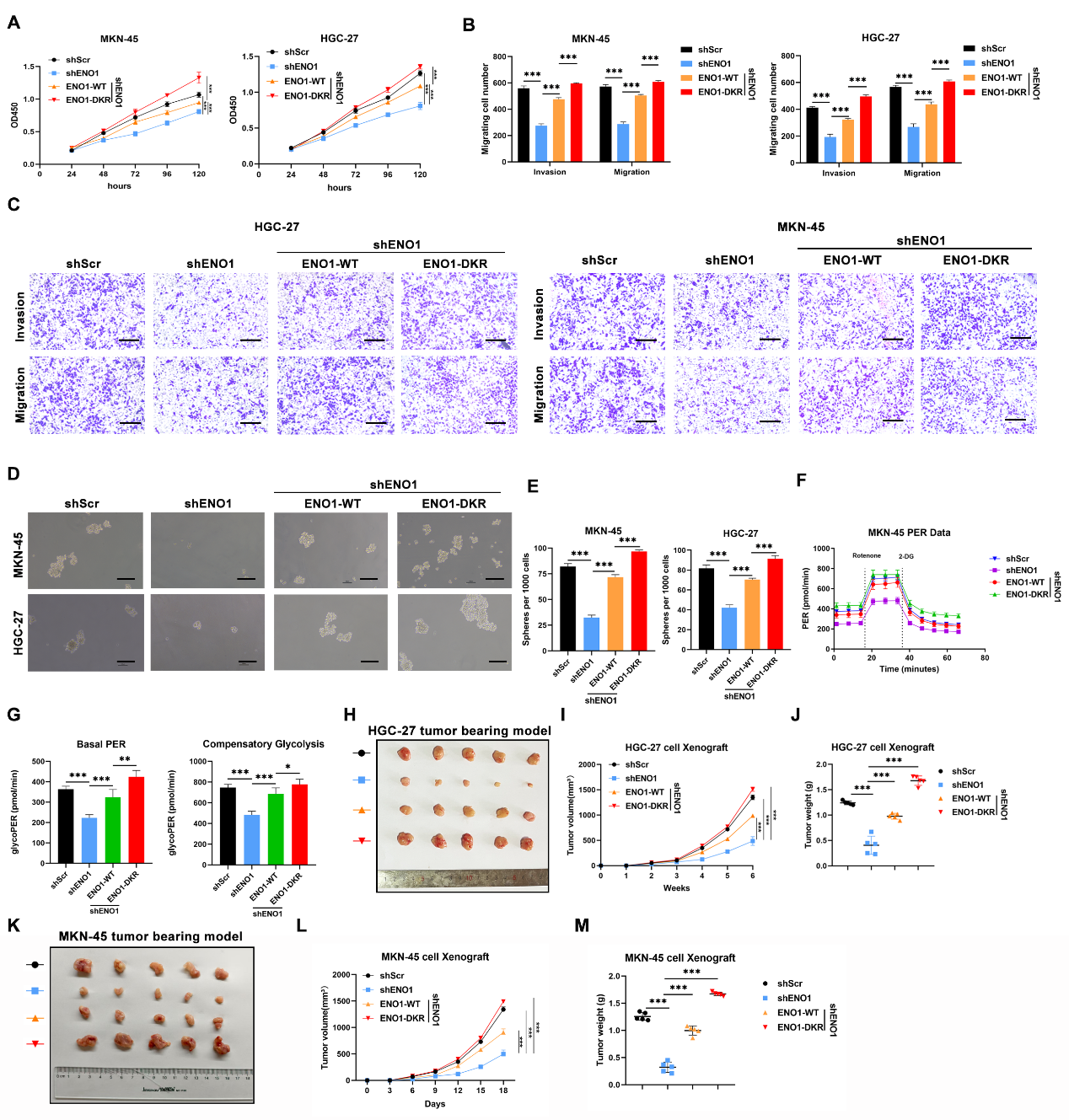


**Fig. S7 SUMO-defective ENO1 contributes to GC cell proliferation and tumorigenesis.**

**A** Cell proliferation ability of GC cells was determined by CCK8 assays. GC cells stably expressed different mutant forms of ENO1 as indicated, instead of endogenous ENO1. **B-C** Cell invasion and migration abilities of GC cells was determined by Transwell assays. GC cells stably expressed different mutant forms of ENO1 as indicated, instead of endogenous ENO1. **D-E** Representative images of formatted spheres among indicated cells. Scale bar, 100μm. **F-G** MKN-45 cells as described cells were measured by the Glycolytic Rate Assay Kit to determine glycolytic proton efflux rate (PER). **H** The images of dissected tumors from HGC-27 cells stably transfected as indicated. **I-J** Tumor weights and sizes are represented as means of tumor weights (J)/sizes (I) ± standard deviation (SD). **K** The images of dissected tumors from MKN-45 cells stably transfected as indicated. **L-M** Tumor weights and sizes are represented as means of tumor weights (M)/sizes (L) ± standard deviation (SD).*p <0.05; **p < 0.01; ***p < 0.001.


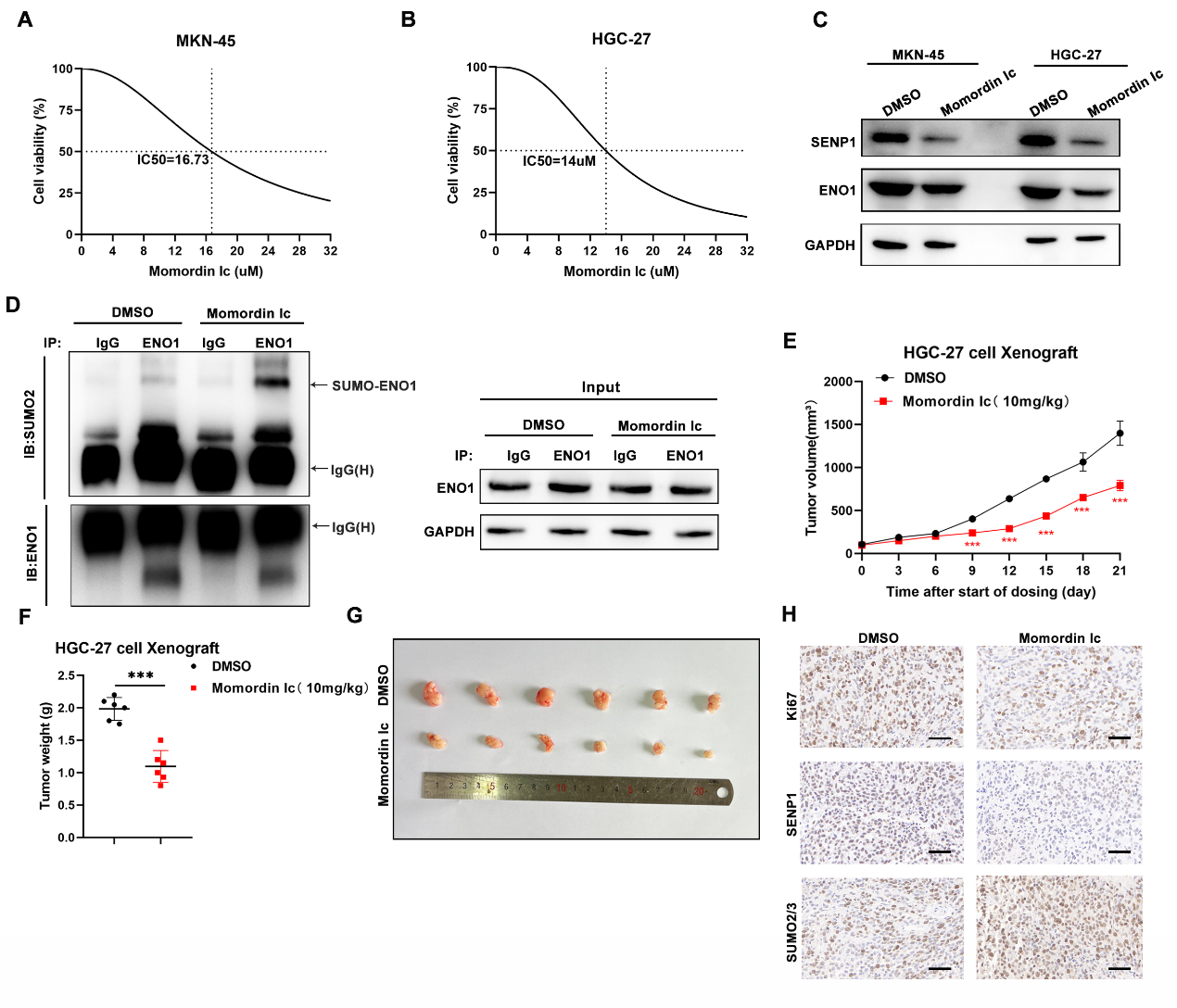


**Fig. S8 Momordin Ic suppresses the GC cell growth.**

**A-B** The sensibility of Momordin Ic of GC cells was measured by CCK8 assay. **C** The protein levels of SENP1 and ENO1 with or without Momordin Ic (16.7 μM in MKN-45 cells and 14 μM in HGC-27 cells) were measured by western blotting. **D** Momordin Ic treatment (16.7 μM in MKN-45 cells and 14 μM in HGC-27 cells) decreased endogenous ENO1 SUMOylation. SUMO band was detected by immunoprecipitation with IgG or anti-ENO1 antibody and then western blotting with anti-SUMO2 antibodies. **E-G** Tumor growth was compared between xenograft nude mice injected with tumor treated with Momordin Ic (10 mg/kg). **H** Representative images of Ki67, SENP1 and SUMO2/3 in subcutaneous tumors of xenograft nude mice by IHC staining. Scale bar, 50 μm. *p <0.05; **p < 0.01; ***p < 0.001.

**Supplementary Table S1. Primers for qPCR**

| **qPCR Primers** | | |
| --- | --- | --- |
| Gene | | Sequence (5’to 3’) |
| SENP1 | F | CGGTTCCGGTTCGGACTTTG |
|  | R | TCGCCTGAGCCAAGAAAACT |
| GAPDH | F | GGGAGCCAAAAGGGTCAT |
|  | R | GAGTCCTTCCACGATACCAA |
| SENP1 promoter | F | CTTGCCAGAGTCTAGCCTAATCC |
|  | R | CTGCACCACCTCATACTTGATTG |
| EP300 | F | GCTTCAGACAAGTCTTGGCAT |
|  | R | ACTACCAGATCGCAGCAATTC |
| ENO1 | F | AAAGCTGGTGCCGTTGAGAA |
|  | R | GGTTGTGGTAAACCTCTGCTC |

**Supplementary Table S2. The shRNA sequences in the study.**

| For shRNA construction |  |
| --- | --- |
| shSENP1-1 | 5'-CAAGAAGTGCAGCTTATAATT-3' |
| shSENP1-2 | 5'-GCAGTGAAACGTTGGACAATT-3' |
| shENO1-1 | 5’-CGTGAACGAGAAGTCCTGCAA-3’ |
| shENO1-2 | 5’-GCCAGCATACTCATCAGTAAT-3’ |
| sip300-1 | 5’-GGAUUCGUCUGUAUGGCUGGUUUAA-3’ |
| sip300-2 | 5’-GCAGCUCAACCAUCCACUATT-3’ |

**Supplementary Table S3. Antibodies**

| **Antibody** | **Host** | **Supplier** | **Catalog No.** | **Application** |
| --- | --- | --- | --- | --- |
| SENP1 | Rabbit | CST | #11929 | WB |
| SENP1 | Rabbit | Abcam | EPR3844 | IP, IHC, IF |
| ENO1 | Rabbit | CST | #3810 | WB, IP |
| ENO1 | Rabbit | Proteintech | 11204-1-AP | IHC, IF |
| SUMO1 | Rabbit | CST | #4930 | WB |
| SUMO2/3 | Rabbit | CST | #4971 | WB, IHC |
| HA | Rabbit | CST | #3724 | WB, IP |
| Flag | Rabbit | CST | #14793 | WB, IP |
| HIS | Rabbit | CST | #2365 | WB, IP |
| Ki67 | Mouse | Servicebio | GB121141-100 | IHC |
| p300 | Rabbit | CST | #54062 | WB, ChIP |
| H3K27ac | Rabbit | CST | #8173 | WB, ChIP |
| GAPDH | Mouse | CST | D4C6R | WB |
